# The insular herpetofauna of Mexico: Composition, conservation, and biogeographic patterns

**DOI:** 10.1002/ece3.7513

**Published:** 2021-04-04

**Authors:** Juan Valentín Pliego‐Sánchez, Christopher Blair, Aníbal H. Díaz de la Vega‐Pérez, Víctor H. Jiménez‐Arcos

**Affiliations:** ^1^ Laboratorio de Herpetología Vivario FES Iztacala Universidad Nacional Autónoma de México Tlalnepantla Mexico; ^2^ Department of Biological Sciences New York City College of Technology The City University of New York Brooklyn NY USA; ^3^ Biology PhD Program, Graduate Center New York NY USA; ^4^ Consejo Nacional de Ciencia y Tecnología‐Centro Tlaxcala de Biología de la Conducta Universidad Autónoma de Tlaxcala Tlaxcala Mexico; ^5^ Naturam Sequi AC Naucalpan Mexico Mexico

**Keywords:** conservation status, exotic alien species, island biogeography, island conservation, island threats, taxonomic turnover

## Abstract

We compile a Mexican insular herpetofaunal checklist to estimate endemism, conservation status, island threats, net taxonomic turnover among six biogeographic provinces belonging to the Nearctic and Neotropical regions, and the relationships between island area and mainland distance versus species richness. We compile a checklist of insular herpetofaunal through performing a literature and collection review. We define the conservation status according to conservation Mexican law, the Red List of International Union for Conservation of Nature, and Environmental Vulnerability Scores. We determine threat percentages on islands according to the 11 major classes of threats to biodiversity. We estimate the net taxonomic turnover with beta diversity analysis between the Nearctic and Neotropical provinces. The Mexican insular herpetofauna is composed of 18 amphibian species, 204 species with 101 subspecies of reptiles, and 263 taxa in total. Endemism levels are 11.76% in amphibians, 53.57% in reptiles, and 27.91% being insular endemic taxa. Two conservation status systems classify the species at high extinction risk, while the remaining system suggests less concern. However, all systems indicate species lacking assessment. Human activities and exotic alien species are present on 60% of 131 islands. The taxonomic turnover value is high (0.89), with a clear herpetofaunal differentiation between the two biogeographic regions. The species–area and species–mainland distance relationships are positive. Insular herpetofauna faces a high percentage of threats, with the Neotropical provinces more heavily impacted. It is urgent to explore the remaining islands (3,079 islands) and better incorporate insular populations and species in ecological, evolutionary, and systematic studies. In the face of the biodiversity crisis, islands will play a leading role as a model to apply restoration and conservation strategies.

## Graphical abstract

The islands of Mexico are home to almost 20% of the diversity of amphibians and reptiles in the country. The classification systems of extinction risk are contrasting, suggesting reviewing and assessing the species and populations inhabiting islands. We recorded 60% of islands with herpetofaunal records facing global threats to biodiversity.

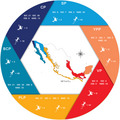

## 1 INTRODUCTION

The islands have served as natural laboratories for the theoretical and empirical study of ecology, evolution, and conservation. Charles Darwin and Alfred R. Wallace were inspired by their observations in the Galapagos Islands and Malayan Archipelago to formulate the theory of evolution by natural selection (Darwin, 1859; Wallace, 1869). Furthermore, the island's systematic studies led from the ecology and biogeography of descriptive work to more analytical approaches through the fundamental book "*The Theory of Island Biogeography*" of Robert MacArthur and Edward Wilson in 1967 (Losos & Parent, 2009). One of the most transcendental conclusions of this book is that the contribution of colonization, extinction, and speciation depends on the island area and the degree of isolation, which together explain species richness (Valente et al., 2020). Thus, island research has provided insights that have fundamentally transformed our view of biogeography, ecology, and evolution (Lomolino et al., 2009).

The oceanic islands are located in all latitudes and contain a considerable proportion of the planet's biodiversity. Due to their geographic isolation, islands harbor a disproportionately high percentage of endemic species, but moderate species richness compared with the mainland (Whittaker & Fernández‐Palacios, 2007). Moreover, islands have been involved in the evolution of both exceptional evolutionary patterns (*e.g*., adaptive radiations, evolutionary convergences) and unique phenotypic and functional traits (Kier et al., 2009; Russell & Kueffer, 2019). Currently, in the face of the biodiversity crisis, island ecosystems are the most threatened (Leclerc et al., 2020; Russell & Kueffer, 2019). Globally, islands are recognized as the epicenter of biodiversity loss (Spatz et al., 2017), with almost 40% of the species extinctions occurring on these systems, directly linked to human activities.

Both natural phenomena and anthropogenic activities can devastate entire island ecosystems. Hurricanes or cyclones have an intense effect on island biota, but these are agents of natural selection that shape the dynamics of colonization and extinction, and to which the species have been exposed and presumably adapted (Donihue et al., 2018; Losos & Ricklefs, 2009). In contrast, human activities are the more critical changing agent threatening the unique island biodiversity due to its short‐time action (Donlan & Wilcox, 2008; Leclerc et al., 2018). Biological invasions, wildlife exploitation, and cultivation have been linked to the majority of insular extinctions and remain as the main threats to extant island species (Donlan & Wilcox, 2008; Leclerc et al., 2018). Additional synergistic factors such as pollution, urbanization, and climate change could accelerate the extinction of insular populations or species (Leclerc et al., 2020).

The risk of extinction of island species depends on both exposure and the interactions between threats (Leclerc et al., 2018). According to the Red List of the International Union for Conservation of Nature (IUCN), amphibians and reptiles are the most under‐assessed groups within the terrestrial vertebrates, with 73% and 87% of evaluated species, respectively, compared with birds (100%) and mammals (91%; IUCN, 2021). The relative scarcity of extinction risk assessments could be due to comparatively limited geographic distribution, ecology, life history, and taxonomic studies (Böhm et al., 2013; Meiri & Chapple, 2016; da Silva et al., 2020; Tonini et al., 2016; Winter et al., 2016). Therefore, meetings continue to complete assessments to more accurately determine the category of risk of extinction (Gumbs et al., 2020; Tonini et al., 2016; Winter et al., 2016). Furthermore, of the amphibian and reptile species recorded as extinct by the IUCN, 62.86% and 90%, respectively, inhabited islands. Because island biodiversity has been the epicenter of global extinctions (Spatz et al., 2017), these patterns are likely to be reflected at local or regional scales.

The complexity of the Mexican coastal landscape has favored the formation of a large number of islands, making it an ideal site for the study of island biotas. Mexico has 11,122 km of shoreline (without insular territory) in two major coasts. On the east coast is the Atlantic Ocean, where the Gulf of Mexico and the Caribbean Sea share 3,294 km of shoreline. Meanwhile, on the west coast, the Pacific Ocean with the Gulf of California share 7,828 km of shoreline. About 68% of the Mexican continental littoral zone belongs to the coast and islands of the Pacific Ocean and the Gulf of California; the rest of this area (32%) is in the shoreline, islands, and cays of the Gulf of Mexico and the Caribbean Sea (SCINMM, 2014). Specifically, the insular Mexican territory comprises 4,110 elements, which include islands, reefs, and cays, covering an area of 7,559.8 km^2^, which represents 0.0004% of the Mexico territory (SCINMM, 2014). Approximately 78.07% (3,209 islands) correspond to true islands (natural surface of variable land permanently emerged and surrounded by a water matrix), covering 94.2% of the insular surface record, and are located in the marine (where the continental shelf ends toward the sea), marine‐coastal (variable width strip that goes from the coastline to where the continental shelf ends), and coastal (from the coastline and up to a height of 200 m asl; it includes coastal lagoons, estuaries, and other water bodies that communicate permanently, intermittently, directly, or indirectly with the sea; SCINMM, 2014) zones.

Here, we compile the distribution of amphibians and reptiles on islands of Mexico to determine the conservation status following three classification systems, endemicity level, threats associated with human activities, and the presence of invasive alien species on islands. Through a literature and scientific collections review, we compile a herpetofaunal checklist, resulting in the first attempt that integrates all the amphibian and reptile records for the Mexican islands (even among the terrestrial vertebrates). Further, with this inventory, we determine the percentage of different threats on islands, threat differences between biogeographic regions, the net taxonomic turnover associated with the Mexican biogeographic provinces, and the relationships between island–area and island–mainland distance against species richness.

## 2 METHODS

**FIGURE 1:**
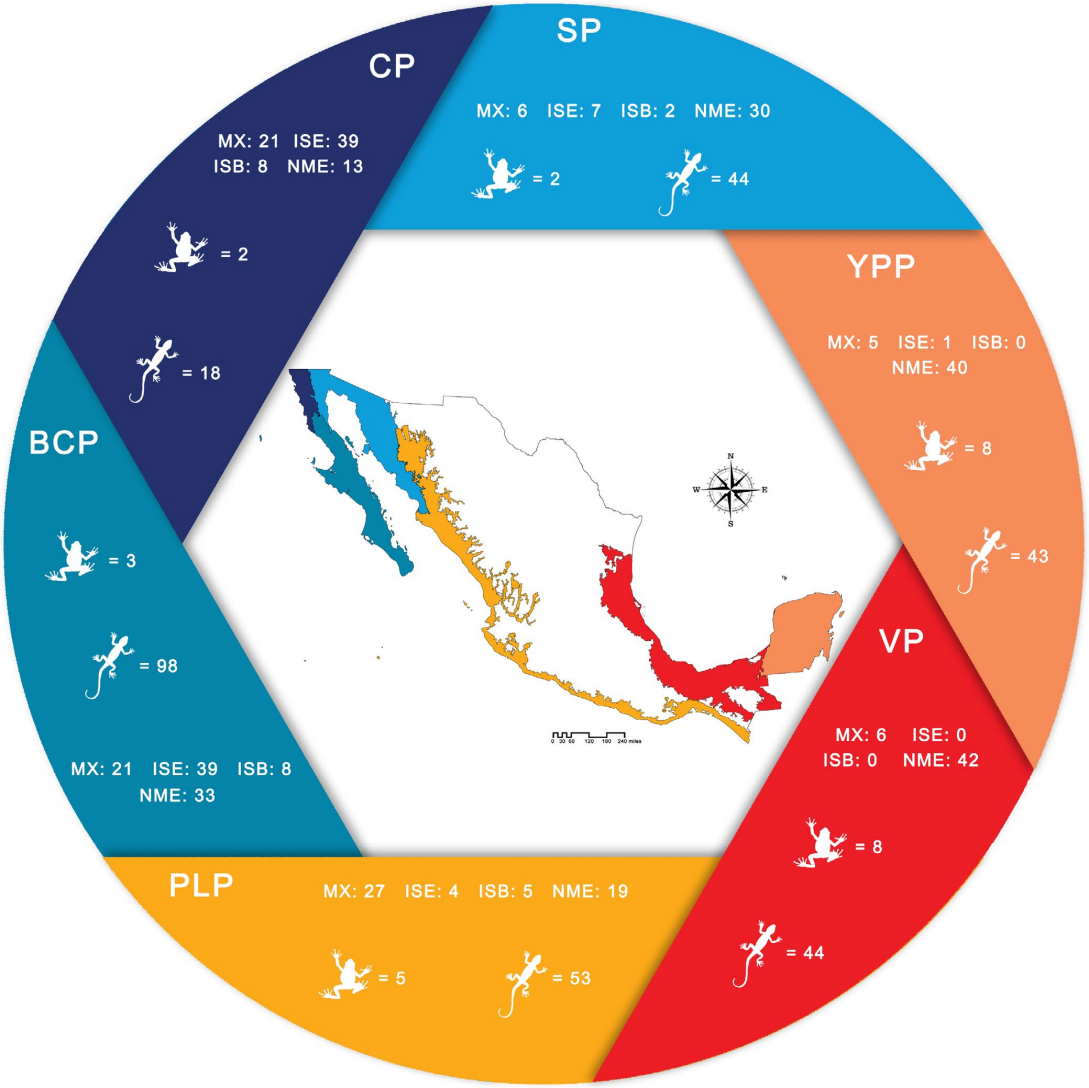
Map of Mexico showing the geographic location of the provinces in the Nearctic (blue colors) and Neotropical (yellow, red, and cream) regions, the number of species endemic to Mexico with a presence on the mainland (MX), island endemic species (ISE), island endemic subspecies (ISB), and nonendemic species (NME). The frog and lizard figures represent total amphibian and reptile species by province. Abbreviations: CP: Californian, BCP: Baja Californian, SP: Sonoran, PLP: Pacific Lowlands, VP: Veracruzan, and YPP: Yucatan Peninsula provinces. The map was based on Morrone et al. (2017)

The islands in Mexico are located in six large oceanic ecoregions (Wilkinson et al., 2009). However, because most amphibian and reptile species have colonized islands from the mainland (to the exception of five marine turtles and one marine snake), we used the Mexican biogeographic province classification to explore the species presence by region and for taxonomic turnover. Thus, we grouped the islands within six biogeographic provinces associated with the Pacific and Atlantic versants. Moreover, this biogeographic classification includes the largest and most isolated islands in Mexico, according to Morrone et al. (2017). The following provinces were used: Californian (CP), Baja Californian (BCP), Sonoran (SP), Pacific Lowlands (PLP), Veracruzan (VP), and Yucatan Peninsula (YPP) provinces (Figure 1). The Tamaulipas province has islands, but no record of amphibians or reptiles. The CP, BCP, and SP were included in the Nearctic region, and PLP, VP, and YPP correspond to the Neotropical region (Morrone et al., 2017; Figure 1).

*Taxonomic position*. We followed the AmphibiaWeb portal (AmphibiaWeb, 2020; http://amphibiaweb.org) for amphibians and Uetz and Hošek (2020) (http://www.reptile‐database.org) for reptile names. For reptiles, Uetz and Hošek (2020) integrated into the database the subspecies category. We included subspecies because some of them are island endemics; therefore, we considered their recognition important. The taxonomic list is presented in Table S1.

### 2.1 Data collection

We performed a literature review to search all possible "checklist," "inventories," and "new records" for "amphibians" and "reptiles," as well as other common names (*e.g*., frogs, toads, lizards, turtles, tortoise, and snakes) of the Mexican islands. We collected information from the literature by executing searches on Google Scholar and Web of Science (https://webofknowledge.com) using the terms mentioned above plus "island," "islands," "cay," or "reef" with "Mexico." We located 16 studies resulting in a list of 111 islands with herpetofaunal records.

Additionally, we used the online platforms of Global Biodiversity Information Facility (GBIF; htpp://www.gbif.org) and Sistema Nacional de Información Sobre Biodiversidad de Mexico (CONABIO, 2020; http://www.snib.mx) to obtain additional records for museum specimens, as well as literature not published in scientific journals but that may contain regional lists or species list reports. Only those records that provided the deposit collection, voucher number (or photograph), coordinates, and the presence in marine or coastal islands were kept. Some coordinates did not coincide with any island despite the locality names, so these were excluded. With this revision, we compiled a total of 131 islands with herpetofaunal records (Table S2).

### 2.2 Endemicity and conservation status

We classified the species as endemic to Mexico with a mainland presence, endemic island species, endemic island subspecies, and nonendemic species. We determined the conservation status of each species from the Official Mexican Standard No. 059 (NOM059 by the Spanish acronym; SEMARNAT, 2010), the IUCN Red List 2020 (IUCN, 2021), and Environmental Vulnerability Scores (EVS; Johnson et al., 2017; Wilson et al., 2013a, 2013b). The NOM059 is a Mexican law that establishes the floral and faunal categories for protection, which includes the following: special protection (Pr), threatened (A), endangered (P), and probably extinct in the wild (E). The IUCN Red List is a classification system widely used in scientific research (e.g., Leclerc et al., 2018; Spatz et al., 2017). The categories include not evaluated (NE), data deficient (DD), least concern (LC), near threatened (NT), vulnerable (VU), endangered (EN), critically endangered (CR), extinct in the wild (EW), and extinct (EX). The last two categories were not recorded in this study. The most threatening categories for nonextinct species include VU, EN, and CR.

The EVS was initially proposed to assess the conservation status of amphibians and reptiles in Mesoamerica and later fitted to the Mexican herpetofauna. It consists of a series of ecological attributes, geographic distribution, reproductive biology (amphibians only), and human persecution level (reptiles only) to determine any of three risk categories: low, medium, and high (see Johnson et al., 2015; Wilson et al., 2013a, 2013b), with subsequent updates (García‐Padilla et al., 2020).

For the three classificatory systems, we determined the status at the species level. The NOM059 is the only system that considers some subspecies under distinct threat categories. Only *Aspidoscelis hyperythrus schmidti* differed with respect to the nominal species, so it was recognized in the analysis. We excluded all invasive alien herpetofaunal species for the percentage estimation.

### 2.3 Islands threats

We determined the presence of the 11 major classes of most significant threats to biodiversity according to the IUCN Red List, based on Leclerc et al. (2018). The definition is presented in Table S3. We determined threats on islands, rather than species, because some herpetofaunal taxa have not been assessed by IUCN, and insular populations could be exposed to different selective pressures (natural and anthropogenic) than continental populations. Furthermore, determining the presence of human activities and invasive alien species on the islands provides a risk assessment for any biological group present on these islands. Of the 11 threats, we considered climate change and geological events as present on all islands. Climate change can generate adverse effects on a global scale (e.g., generating drought, increasing the magnitude of climatological phenomena, or the complete sinking of islands), which could threaten the permanence of amphibians and reptiles on islands (Bellard et al., 2014; Winter et al., 2016). In the case of geological events, Mexico is located in the Ring of Fire, an area of high seismic and volcanic activity (García Acosta, 2004), which may increase the probability of catastrophic geological events on islands. For wildlife exploitation, we assume that any island with endemic insular species (in our case, all were reptiles) is subject to this threat, based on the fact that reptiles are the most trafficked legally and illegally worldwide (D’Cruze & Macdonald, 2016). Because these first three threats can obscure other human activities that endanger the insular herpetofauna or could be speculative (as in wildlife exploitation), we model two scenarios. Scenario 1 includes the climate change, geological events, and wildlife exploitation active. Therefore, in Scenario 2 we do not consider these threats.

For the remaining threats, we conducted a search for human settlements and activities (e.g., cultivation, permanent human population, tourism, seasonal fishing, mining/natural resources extraction), as well as the presence of invasive alien species. For human settlements and activities, we obtained information from the Atlas of the Inhabited Insular Territory of Mexico (INEGI, 1994). We used the invasive species diagnosis in protected natural areas of Mexico by the National Commission for the Knowledge and Use of Biodiversity (Aguirre‐Muñoz et al., 2013) and the Threatened Island Biodiversity Database Partners (2018) (available in http://tib.islandconservation.org.) to determine the presence of invasive plants and animals. The first and second documents provide information on human activities such as fishing, agriculture, livestock, mining, and tourism developed on the islands. The Threatened Island Biodiversity Database provides the presence of invasive vertebrates on 60 Mexican islands. Although some groups of invasive alien species (e.g., vertebrates as rats or cats) may pose more significant threats than others (e.g., plants), we consider any introduced plant or animal as invasive, because their presence could alter the island ecosystem dynamics (Russell & Kaiser‐Bunbury, 2019). The treefrog *Trachycephalus typhonius* and the snake *Boa imperator* are considered invasive alien species on Isla Cozumel (Martínez‐Morales & Cuarón, 1999; Pavon‐Vazquez et al., 2016). However, since these species are naturally distributed in the mainland and islands of Mexico, we considered them as part of the insular herpetofauna. The threats recorded on each island are shown in Table S4.

*Island threats between biogeographic regions*. To determine differences in the threats between Nearctic and Neotropical regions, we used a Mann–Whitney *U* test, using the regions as category and the threat numbers recorded by island as dependent variable. We selected this test because normality and homoskedasticity failed even when different transformations were used. Also, because the number of islands for some provinces is low (VP: eight islands; PC: seven islands), it was not possible to determine differences between provinces using other statistic methods. The analysis was carried out with the R program version 4.0.1 (R Core Team, 2020), considering an alpha value of 0.05.

### 2.4 Taxonomic turnover

The taxonomic turnover between biogeographic provinces was assessed with the Simpson dissimilarity index (βsim). We used this index because it allowed us to identify the role of the unshared biota size components in β diversity analyses. Otherwise, it would enable estimating the net taxonomic turnover between biotas (Baselga, 2010; Baselga & Orme, 2012). Analyses were performed with R version 4.0.0 (R Development Core Team, 2020) with the “betapart” package (Baselga & Orme, 2012). We emphasize that results from this analysis may be an underestimate because, for some islands, there are no systematic studies to determine taxonomic diversity.

### 2.5 Area and continental distance versus species richness

Several mathematical models have been used to infer the relationship between area and species richness (Triantis et al., 2012). We used the power model since it is the simplest and widely utilized (Triantis et al., 2012). We performed a natural logarithm (Ln) transformation of island area, continental distance, and species richness. This transformation enabled us to estimate the model parameters with linear regression analysis (Preston, 1962). Island area and mainland distance (the minimum distance from the closest end of the island to the mainland) were the independent variables, and species richness was the dependent variable. The analysis was carried out with the R program version 4.0.1 (R Core Team, 2020), considering an alpha value of 0.05.

## 3 RESULTS

We found that the insular herpetofauna was composed of 222 species with 101 subspecies among 131 islands. The number of subspecies ranged from one to six, with 263 total taxa recognized (Table 1; Table S3). Species richness was markedly unequal between amphibians and reptiles. For amphibians, we recorded only 18 species, belonging to two orders with two salamanders and 16 frogs on 22 islands. For reptiles, we found 204 species and 101 subspecies. All major reptile groups were represented on islands, including two crocodiles, one worm lizard, 128 lizards, 99 snakes, 14 turtles, and one tortoise species and subspecies, on a total of 130 islands.

**TABLE 1:** Taxonomic summary of the Mexican insular herpetofauna by biogeographic province and region

	Orders		Suborders		Families		Genera		Species		Subspecies		Total Taxa		
	A	R	A	R	A	R	A	R	A	R	A	R	A	R	H
CP	1	1	‐	2	1	6	2	12	2	14	‐	13	2	18	20
BCP	1	2	‐	3	3	13	3	31	3	82	‐	34	3	98	101
SP	2	2	‐	2	2	11	2	25	2	40	‐	22	2	44	46
Nearctic	**2**	**2**	**‐**	**3**	**4**	**16**	**5**	**36**	**5**	**111**	**‐**	**56**	**5**	**138**	**143**
PLP	1	3	‐	2	4	15	5	35	5	46	‐	23	5	53	58
VP	1	3	‐	2	5	16	7	34	8	43	‐	18	8	44	52
YPP	1	3	‐	2	4	21	8	32	8	42	‐	11	8	43	51
Neotropical	**1**	**3**	**‐**	**2**	**6**	**25**	**10**	**60**	**13**	**97**	**‐**	**47**	**13**	**110**	**123**
Total	**2**	**3**	**‐**	**3**	**8**	**29**	**15**	**81**	**18**	**204**	**‐**	**100**	**18**	**245**	**263**

Abbreviations

A, amphibians; BCP, Baja Californian; CP, Californian; H, herpetofauna; PLP, Pacific Lowlands; R, Reptiles; SP, Sonoran; VP, Veracruzan; YPP, Yucatan Peninsula provinces.

The richness of the taxonomic groups of order, suborder (reptiles only), family, genus, species, and subspecies (reptiles only) is indicated. Numbers in bold means the total taxonomic groups for each biogeographic regions and the total insular Mexican herpetofauna.

One to eight amphibian species were recorded per island (mean ± SE; 2.04 ± 0.39). In the Neotropical region, we recorded 12 islands (five islands in YPP, four in PLP, and three in VP) with 13 of the 18 amphibian species. The Nearctic provinces harbored five species on 10 islands (five islands in BCP, four in CP, and one in SP). For reptiles, the species number ranged from one (39 islands) to 37 species (Isla del Carmen, Campeche) per island (5.98 ± 0.59) for both biogeographic regions. In contrast with amphibians, the pattern was reversed in reptiles, with the Nearctic region harboring the highest species richness with 139 taxa among 90 islands (5.34 ± 0.61). The Neotropical region was home to 125 species and subspecies on 40 islands, showing on average more species per island (7.24 ± 1.27).

For amphibians and reptiles in sum, the mean species number per island was 6.28 ± 0.65. The BCP showed the highest species richness with 101 taxa, and CP had the lowest number with 20 species and subspecies (Table 1; Figure 1). Most island records corresponded to the BCP (69 islands), followed by the PLP (21 islands), SP (14 islands), YPP (12 islands), VP (8 islands), and CP (7 islands). Island number was also greater in the Nearctic (90 islands) versus Neotropical (40 islands) zone. When we compared the insular surface area, it was almost three times greater in the Nearctic (76.03%; 3,744.15 km^2^) than in the Neotropical (23.97%; 1,180.34 km^2^). The relatively large Isla Tiburon, located in the SP, had a larger surface area (1,198.75 km^2^) than all the insular bodies combined for the Neotropical region. However, Isla del Carmen, Campeche, located in the Neotropical region in the VP, showed the greatest species richness (six amphibians and 37 reptiles). This island has an area less than 12% of Isla Tiburon, but showed three times more amphibian and 11 more reptile taxa. For 40 islands, we found only one species record (one and 39 for amphibians and reptiles, respectively). The smallest island with records was Cayo Lobos, Quintana Roo (0.003 km^2^). The average of the island surface area was 37.59 ± 12.68 km^2^. The closest island to the mainland was Isla Willard, Baja California (0.03 km), the farthest was Isla Clarion, Colima (702 km), and the average mainland distance was 27.74 ± 7.20 km.

### 3.1 Endemicity and conservation status

Of the 17 native amphibian species, only two (11.76%) are endemic to Mexico, and no species was an island endemic. For reptiles, 53.75% (240 taxa) were endemic to Mexico. Of this percentage, 25.83% (61 species and subspecies) represented endemic species with mainland presence, 20.83% (50 species) are endemic island species, and 7.08% (17 taxa) are island endemic subspecies. The distribution of endemic insular species and subspecies was not homogeneous among the provinces of Mexico. The provinces in the Nearctic region contained 45 species and 12 subspecies (57 taxa; Figure 1). These endemic taxa were recorded on 44 islands, with one to eight endemic taxa per island (2.23 ± 0.22), breed on an average of 1.81 islands (range 1 to 12 islands), and 71.93% inhabited only one island (41 taxa). The average island area was 76.53 ± 32.28 km^2^ (range 0.004 to 1,198.75 km^2^), and the mean mainland distance was 13.60 ± 1.55 km (range 1.33 to 51.00 km).

For the Neotropical provinces, the endemic insular taxa were grouped in PLP (four species and five subspecies) and YPP (one species), inhabiting 11 islands with one to three taxa per island (1.83 ± 0.26) (Figure 1). Approximately 45.45% bred on one island (five taxa); the average area was 79.21 ± 42.03 km^2^ (range 0.12 to 467.89 km^2^), and the average mainland distance was 157.49 ± 73.21 km (range 1.78 to 700 km). The endemic island species were represented by 34 lizards and 16 snakes, and the subspecies included seven lizards and ten snakes. Nonendemic species represented 46.25% (111 taxa) of the total number of taxa. We recorded one invasive alien species of frog (*Eleutherodactylus planirostris*) and five species for reptiles, including four lizards (*Anolis sagrei*, *Gehyra mutilata*, *Hemidactylus frenatus*, and *H. turcicus*) and one snake (*Indotyphlops bramminus*). All the invasive herpetofauna were recorded in the provinces of the Neotropical region.

**FIGURE 2:**
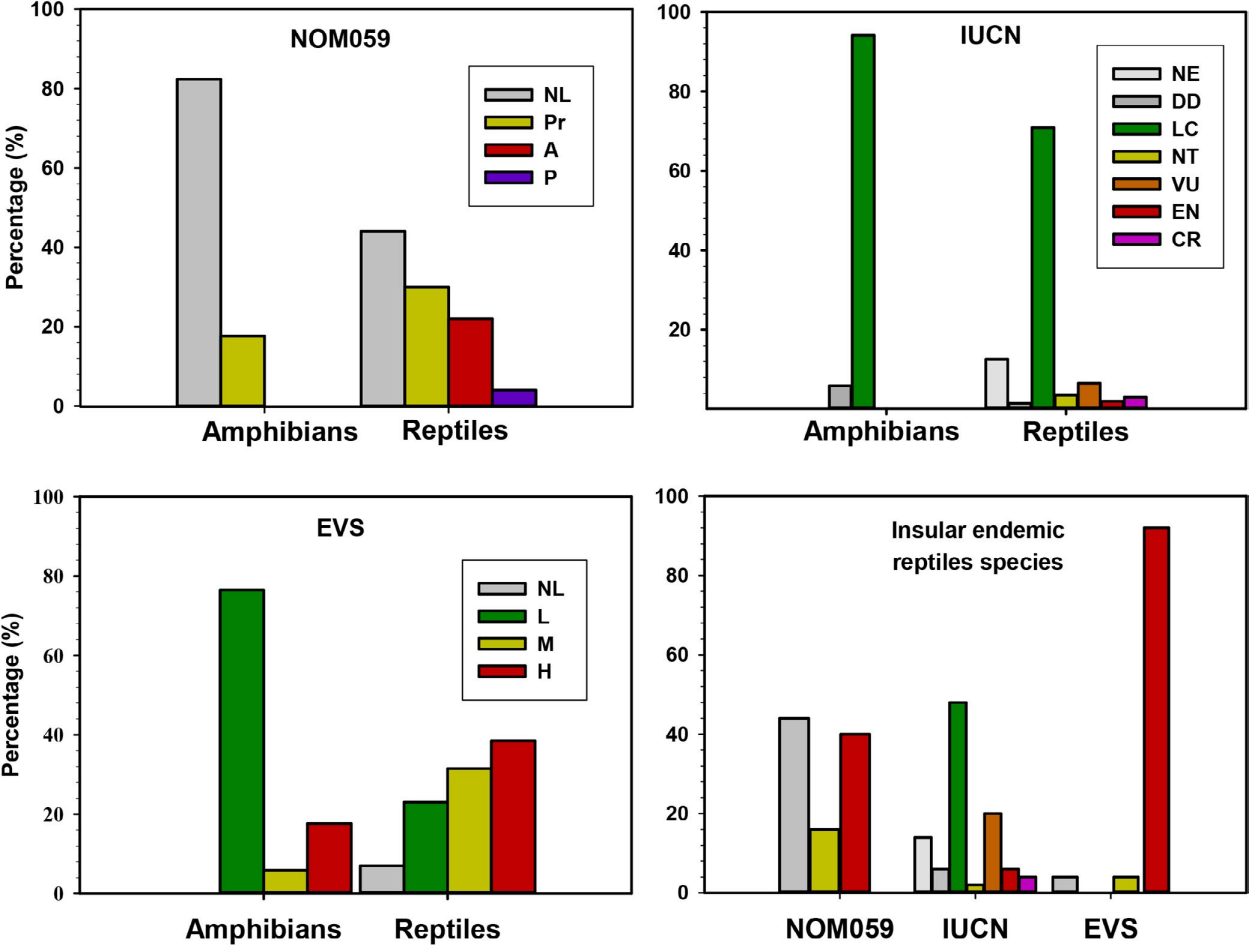
The percentages of conservation status according to the three classification systems: NOM059, IUCN Red List, and EVS. The bottom right shows the status exclusively for endemic island species (reptiles only) under these three systems. Abbreviations: NL: not listed, Pr: special protection, A: threatened, P: danger of extinction, NE: not evaluated, DD: data deficient, LC: least concern, NT: near threatened, VU: vulnerable, EN: endangered, CR: critically endangered, L: low, M: medium, and H: high‐risk category

According to the NOM059, the majority of amphibians were not listed (82.35%), and only three (17.65%) of the 17 species were classified as a Pr category. For reptiles, 112 species (56%) were grouped into the three of the four evaluation risk categories (Pr: 30%; A:22%; and P: 4%), and 44% were not listed (88 species; Figure 2). For the IUCN Red List, the majority of species for both groups were recorded as LC (amphibians: 94.12%; and reptiles: 70.85%), followed by DD (5.88%, one amphibian species) and NE (12.56%, 25 reptile species; Figure 2). We did not record amphibian species within some high‐risk categories (VU, EN, or CR). The high‐risk categories for reptiles represented 11.56% (23 species), and DD (3 species) represented 5.03% (Figure 2). The EVS showed the lowest unlisted values among the three systems for amphibians (all assigned to some category) and reptiles (7.00%, 14 species; Figure 2). For amphibians, most species (76.47%, 13 species) were in the low vulnerability category. For reptiles, most species were grouped in the high category (38.50%, 77 species), followed by medium (31.50%, 63 species) and low categories (23.00%, 46 species) (Figure 2). When we explored the conservation status only for insular endemic species, the trends were similar. The NOM059 and EVS categorized most island species in the highest risk categories (Figure 2). On the IUCN Red List, 48% of the insular species were classified as LC, 30% in the three high‐risk categories, and the remaining 22% as DD and NE (Figure 2).

### 3.2 Island threats

**FIGURE 3:**
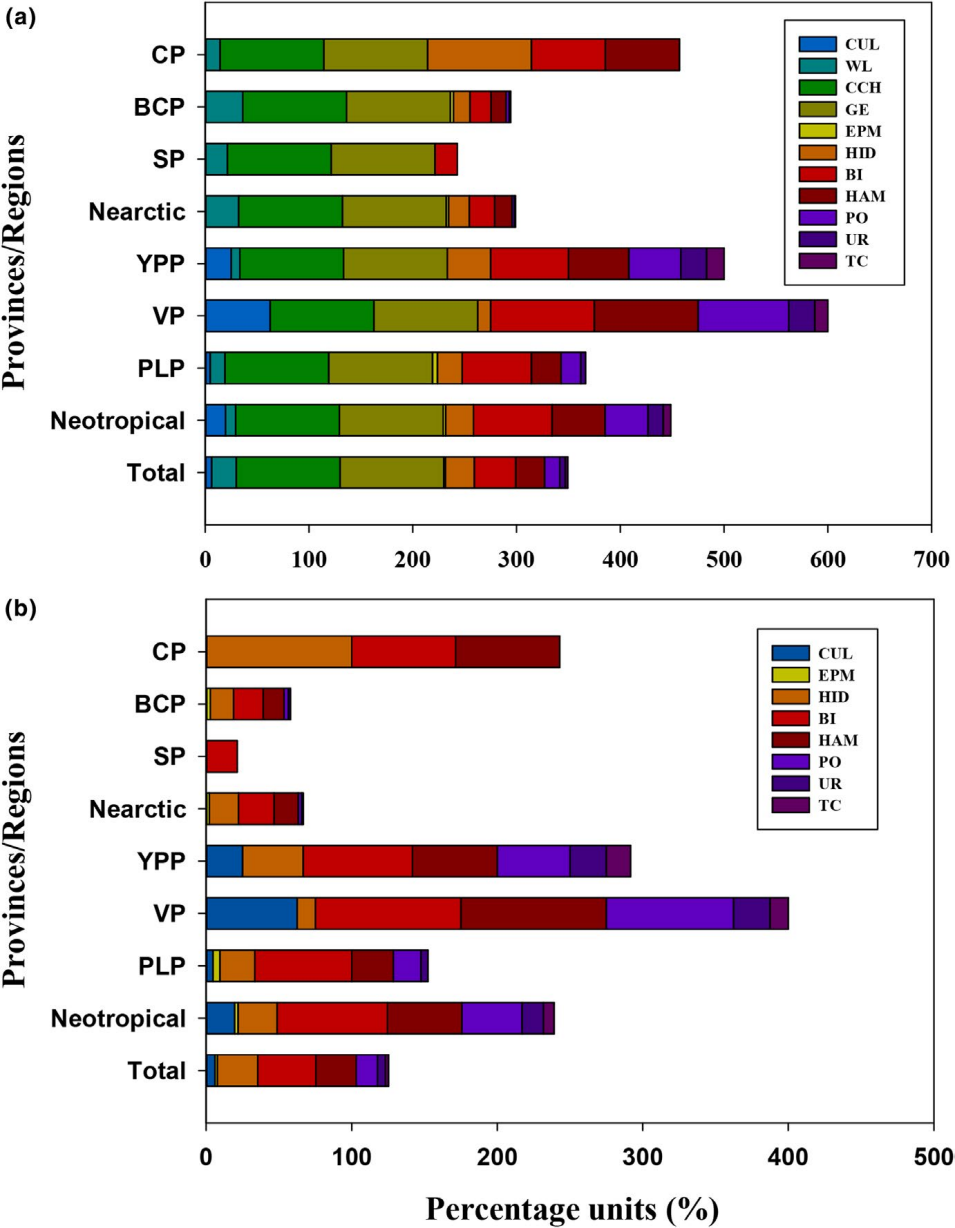
The cumulative percentage of the nine threats associated with human activities (excluding climate change and geological events) by province, region, and total islands. If the bar is larger, the percentage of threats is higher. A) Scenario 1 and B) Scenario 2. Abbreviations: CP: Californian, BCP: Baja Californian, SP: Sonoran, PLP: Pacific Lowlands, VP: Veracruzan, and YPP: Yucatan Peninsula provinces. CUL: cultivation, WL: wildlife exploitation, CCH: climate change, EPM: energy production and mining, GE: geological events, HID: human intrusions and disturbance, BI: biological invasions, HAM: habitat modifications, PO: pollution, UR: urbanization, and TC: transport corridors

With the first scenario, all islands are threatened by at least two processes, climate change (100%) and geological events (100%). The third greatest threat recorded for all the islands together was biological invasions (40.46%; Figure 3a). In this scenario, wildlife exploitation (25.19%) was the fifth threat recorded for all the islands. The second scenario, the most conservative, suggests that the main threats were biological invasions (40.46%), habitat modifications (27.48%), and human intrusions and disturbance (22.14%; Figure 3b). The threats by the biogeographic regions were distinctive. For the Nearctic region (Scenario 1), wildlife exploitation (32.22%) was the first major threat (Figure 3a). In Scenario 2, biological invasions (24.44%), human intrusions and disturbance (20.00%), and habitat modifications (16.67%) were the main threats that had the highest percentage (Figure 3b). In the Neotropical region (Scenario 1), the third greatest threat was biological invasions (75.61%; Figure 3a). For Scenario 2, biological invasions, habitat modifications (51.22%), and pollution (41.46%) were mostly observed (Figure 3b). We recorded significant differences between the biogeographic regions, where the Neotropical region had significantly greater threats both in Scenario 1 (*U* = 3,392.00, *p <.0001*) and in Scenario 2 (*U* = 3,392.00, *p <.0001*). The biogeographic provinces with the highest percentages of registered threats were VP, YPP, and CP in both scenarios (Figure 3a, b).

When we explored threats on islands inhabited by amphibians, the greatest threats were biological invasions (90.91%), human intrusions and disturbance (63.64%), and habitat modifications (63.64%) in the first and second scenarios. For amphibians in the Nearctic region, human intrusions and disturbance (80%), biological invasions (80%), and wildlife exploitation (80%) remained as the main threats in Scenario 1, and habitat modifications (60.00%) as the third major threat in Scenario 2 when wildlife exploitation is excluded. For the Neotropical region, the first two threats remained identical, increasing the percentage in both biological invasions (100%) and human intrusions and disturbance (66.67%), and pollution (58.33%) was the third largest threat on record in the first and second scenarios. Because reptiles were recorded on 130 of the 131 islands, the threat percentages remained very similar to the general pattern in both scenarios and all taxa.

### 3.3 Taxonomic turnover

**FIGURE 4:**
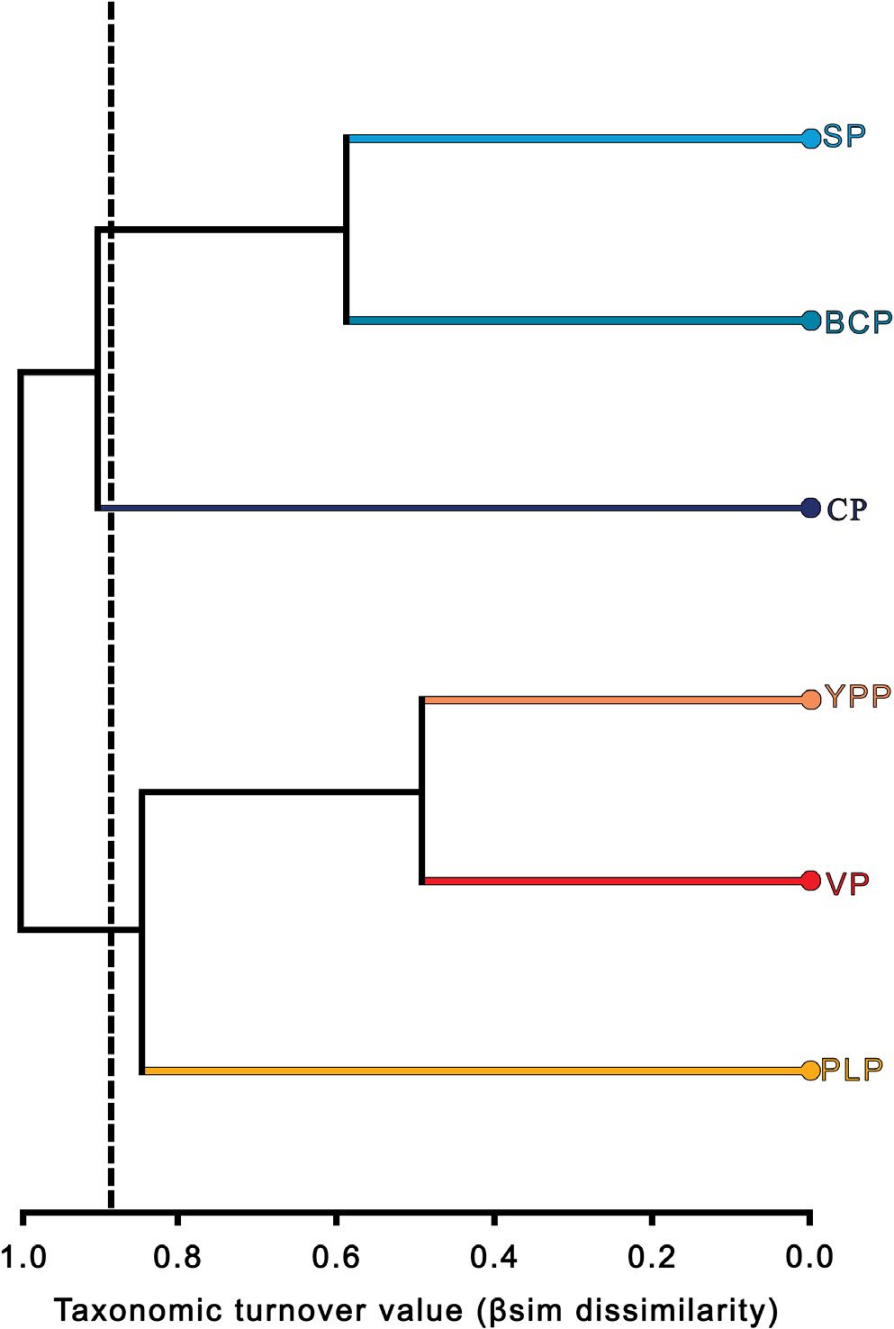
Classification analysis based on dissimilarity by net taxonomic turnover of the insular herpetofauna of Mexico among the biogeographic provinces. The βsim index and the complete ligation method were used. The dotted vertical line indicates the average value of the dissimilarity matrix (0.89). Abbreviations: CP: Californian, BCP: Baja Californian, SP: Sonoran, PLP: Pacific Lowlands, VP: Veracruzan, and YPP: Yucatan Peninsula provinces. Branches are colored according to Figure 1 provinces

The average regional taxonomic turnover (β_sim_) value was 0.89 ± 0.16 (mean ± SD). The BCP and PLP showed the highest number of unique species and subspecies (58.45%; Table 2). The lowest dissimilarity values corresponded to VP‐YPP and BCP‐SP (Table 2). Considering only the unique species and subspecies by provinces (107 taxa), the net taxonomic turnover was 40.46%. The classification analysis based on dissimilarity was comprised of two main groups: one integrated by the Nearctic (CP, BCP, and SP) and the second by the Neotropical (PLP, VP, and YPP; Figure 4) provinces. For the Nearctic group, we recorded 139 taxa not shared. The BCP and SP formed a subgroup, defined by a lower dissimilarity (0.50) given the account of shared species. For the Neotropical zone, 119 species and subspecies were unique to the three provinces in this group. Similarly, the subgroup formed by VP and YPP showed less dissimilarity (0.49) due to more shared taxa (Figure 4). Nearctic and Neotropical regions shared four species; three were shared between two provinces: one marine turtle (*Lepidochelys olivacea*; BCP‐PLP) and two lizards (*Sceloporus clarkii clarkii* and *Urosaurus ornatus schotti*; SP‐PLP); and one was shared among PLP, BCP, and SP (*Crotalus atrox*).

**TABLE 2:** The number of unique taxa (not shared) per biogeographic province is shown on the main diagonal (bold). Below the diagonal are the net taxonomic turnover values, above the diagonal are a number of species shared between provinces, and number in parentheses indicates taxa shared among three provinces

	CP	BCP	SP	PLP	VP	YPP
CP	**15**	*3* (*1*)	*1* (*1*)	*0*	*0*	*0*
BCP	0.80	**78**	*17* (*1*)	*1* (*1*)	*0*	*0*
SP	0.90	0.59	**24**	*2* (*1*)	*0*	*0*
PLP	1.00	0.96	0.93	**43**	*1* (*7*)	*3* (*7*)
VP	1.00	1.00	1.00	0.85	**25**	*19* (*7*)
YPP	1.00	1.00	1.00	0.80	0.49	**22**

### 3.4 Area and continent distance versus species richness

**FIGURE 5:**
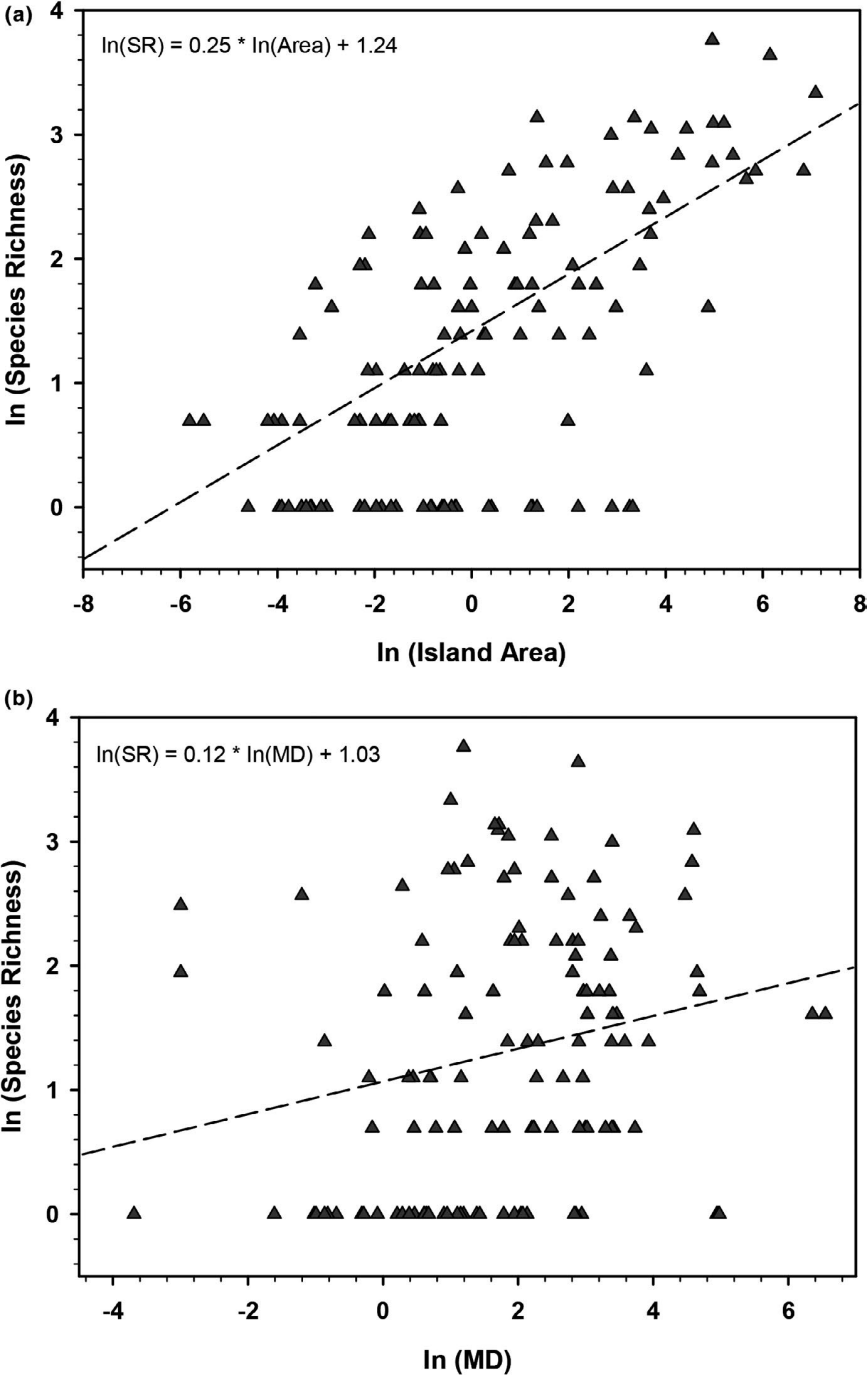
The positive linear relationship between the natural logarithm (Ln) of the A) island area and B) mainland distance (MD) against species richness (SR)

According to theory, we found a significantly positive relationship between island area and species richness (*F*_1,128_ = 88.69, *p <*.0001). The model explained 40.28% of the variance (Figure 5a). Contrary to what we expected, we found a significant increase in species number with the mainland distance (*F*_1,128_ = 5.61, *p =.019*), although the percentage of explained variance was lower (3.43%; Figure 5b).

## 4 DISCUSSION

### 4.1 Composition and conservation status

The Mexican herpetofauna is the most diverse in the Mesoamerican region, which is the region that occupies the second place for biodiversity hot spots in the world (Johnson et al., 2017; Myers et al., 2000). Our results suggest that the islands of Mexico harbor 17.18% of amphibian and reptile species recorded for the country (1,292 species; Johnson et al., 2017), with this percentage contained in a small and restricted area (0.0002% of Mexico territory). To commensurate diversity, it must be considered in comparative terms with other highly diverse areas, such as the rainforest of the Biosphere Reserve Los Tuxtlas and the Lacandona region, which harbor 15% and 9.67%, respectively, of Mexican herpetofauna (Hernández‐Ordóñez et al., 2015; López‐Luna, 2017). Considering that the sampled insular area represents 65.14% of the entire Mexican insular territory, with 3,079 islands (considering only true islands) left to study, the species richness on islands is likely to increase. Continuing exploration and documentation of taxonomic and functional diversity on islands is a promising avenue of research, which is necessary given the current crisis of biodiversity loss.

The conservation status among the three systems yields conflicting recommendations, especially in reptiles. First, the percentage of unlisted species in the NOM059 (82.35% and 44.00% for amphibians and reptiles, respectively) suggests that these taxa are not at risk of extinction or assessments have not been performed. Both claims are not mutually exclusive, although the second option is likely the most plausible. Currently, the NOM059 is the only legal instrument that establishes the conservation status category for wildlife use, management, and exploitation in Mexico (SEMARNAT, 2010). The species inclusion into some category requires exhaustive reviews by experts from each group, under a lengthy bureaucratic process given its impact in the Mexican legal framework, which could delay species or subspecies incorporation, and therefore its legal protection. Second, the IUCN Red List categorizes most species as least concern (amphibians: 94.12%; and reptiles: 70.85%), even for endemic island species (48%). This could be interpreted as that the majority of species inhabiting islands are not at risk of extinction, despite that 60% of islands register more than three significant threats linked to vertebrate extinctions (Leclerc et al., 2018). It is likely that the high percentage of species categorized as least concern is due to several species having broadly distributed continental populations. The IUCN Red List also does not consider subspecies (although only one subspecies is considered in the NOM059 system) or island populations, which potentially masks the unique threats facing insular herpetofauna. It is also likely that many species harbor distinct insular lineages and evolutionarily significant units (ESUs). Thus, phylogenetic and systematic studies will be vital to appropriately characterize the uniqueness of island populations. We propose to prioritize those species not listed, or at least the 22 island endemic species missing in the NOM059 and the 25 species (seven island endemics) listed as NE by the Red List. As stated above, we also need to establish a distinction between island and mainland populations, which could trigger specific conservation programs for island populations or species. The EVS system groups the insular reptiles into a more top risk category (if the medium and high categories are considered). However, its application is restricted to the herpetological community, and it suffers from the same limitation of not recognizing island populations. It is important to reiterate that in this study, we determine the main threats on islands and not on species. The extinction risk assessments used in classification systems are focused on species, and therefore some elements (*e.g*., ecology, population size) need to be considered in future research of island populations and species. Even intrinsic biological characteristics (*e.g*., body size) may increase the risk of extinction (Slavenko et al., 2016).

The information bias on taxonomy, ecology, and distribution of amphibians and reptiles is also recognized (Meiri & Chapple, 2016; da Silva et al., 2020; Winter et al., 2016), and in developing countries such as Mexico, it can generate an imprecise conservation status evaluation (Koleff et al., 2009). Our results are limited to records on islands that do not result from long‐term studies to determine patterns of diversity; rather, information is derived from historical collections and fortuitous encounters. Our example focuses on Mexico, which is located in one of the most diverse areas in the world. It is possible that the Central and South American countries show similar patterns of information bias for their island biodiversity, as well as possible conflicts between status conservation systems and their specific laws.

### 4.2 Threats to the insular herpetofauna

An interesting aspect of our regional approach is that it allows us to identify differential threat scenarios between the Nearctic and Neotropical regions. The geographic location and the species composition could be associated with these differences. The Nearctic region is drier and colder than the Neotropical region; thus, cultivation is limited, given the extremely low rainfall regimes (Grismer, 2002). This implies that human activities and settlements in Nearctic islands are less frequent and mainly associated with lighthouses, fishing camps, Mexican armed forces, and scientific research stations (INEGI, 1994; Samaniego‐Herrera et al., 2007), with otherwise a low human population. This region harbors the majority of insular endemic species, with several island endemic rattlesnakes (genus *Crotalus*). The rattlesnakes are commercialized in the international pet trade, which includes endemic island species (Avila‐Villegas & View, 2005; Fitzgerald et al., 2004). However, all insular endemic taxa are at risk since reptiles are the most heavily traded vertebrate group around the world (D'Cruze & Macdonald, 2016), which could explain that the more persistent threat for Nearctic provinces was wildlife exploitation (32.22%) on the first scenario. However, in Scenario 2, habitat modifications acquire greater relevance, albeit with a relatively low percentage on the islands due to the reduced human population inhabiting islands in this region.

The islands in the Neotropical region show a more worrying scenario, defined by significantly higher threat percentages. Concordantly on a global scale, biological invasions are the main threat for Neotropical islands (75%). Moreover, this region shows a major predominance of human activity. For example, the island with the highest species richness (Isla Del Carmen, Campeche) contains a city with intense oil activity and a human population of 169,466. Also, the Cozumel (surface: 467.89 km^2^) and Mujeres (3.86 km^2^) islands are some of the leading tourism destinations in Mexico with a human population of 100,000 and 13,315, respectively. Specifically, Isla Cozumel has a great diversity of endemic vertebrate taxa among the Mexican islands, and at the same time exhibits the highest number of invasive alien species, brought to the island by human activities (Martínez‐Morales & Cuarón, 1999; Spatz et al., 2017). The elevated presence of human populations can be associated with stable climatic conditions due to being in the tropical zone and proximity to the mainland, although even more remote islands (e.g., Isla Clarion and Isla María Madre) contain invasive alien species and human settlements (e.g., Isla Socorro and Isla Clarion). Invasive vertebrate species eradications (i.e., rodents or cats) have been successful on some islands in both the Nearctic and Neotropical regions (Aguirre‐Muñoz et al., 2013). However, invasive floral and faunal eradication is still pending on many other islands (e.g., Isla Cozumel). We also wish to highlight that the interaction between threats (e.g., with climate change, pollution) can generate scenarios of considerable adversity for conservation (Leclerc et al., 2018), which requires further investigation.

Overall, biological invasions, human intrusions and disturbance, and habitat modifications are the main threats for the insular herpetofauna. Biological invasions are the only threat associated with species extinctions or population declines that have also been identified on a global scale (Leclerc et al., 2018), and this threat has been suggested as a major cause of insular vertebrate extinctions (Donlan & Wilcox, 2008). We analyze the presence of the threats on islands among all the species, rather than in high‐risk species in isolation (Leclerc et al., 2018; Spatz et al., 2017), which may explain the differences in the relative importance of the threats. Also, differences in scale and methodological approaches lead to differences in the results. Similar scale approximations can provide information for the development of regional‐ or country‐specific conservation strategies. For highly diverse nations, usually developing countries, it is essential for the future conservation of island herpetofauna and biodiversity in general.

### 4.3 Biogeographic patterns

The insular Mexican herpetofauna shows a clear differentiation by biogeographic regions, defined by the high taxonomic turnover and dissimilarity estimations. Even on the Pacific coast, where provinces of both biogeographic regions converge, the species composition of the PLP maintains greater similarity with the Neotropical provinces of the opposite coast. Thus, the herpetofaunal differentiation is likely defined by the colonization of specific lineages linked to a Nearctic or Neotropical origin. In the continental part of Mexico, the overlap of the two biogeographic regions generates the Mexican Transition Zone (MTZ), defined by an extensive biotic complexity and great diversity of species (Morrone, 2020). A plethora of examples in plants and animals explore the biogeographic history of Nearctic or Neotropical taxa and dispersal throughout North, Central, and South America (see Halffter & Morrone, 2017; Morrone, 2020). However, on islands, the MTZ may be less clear, at least for amphibians and reptiles, suggesting a shared biogeographic history among the groups that have colonized the islands. A phylogenetic approach is necessary to better understand the biogeographic and evolutionary patterns and processes associated with the herpetofauna diversity on the islands of Mexico.

The relationship between species richness and insular surface area agrees with the theory. Larger islands potentially hold more resources, ecological niche variation, and a lower extinction rate, which favors a higher species richness (MacArthur & Wilson, 1967); although other factors, such as island age, may be important (see Emerson & Oromi, 2005; Gillespie et al., 2008; Losos & Ricklefs, 2009). In contrast, the positive relationship between the distance to the continent and the number of species is not consistent with what is theoretically expected. Although the percentage of variance explained in our model is low, the significant relationship may be due to islands farthest from the mainland being larger. However, we did not find a significant relationship between area and distance from the continent (data not shown), at least with our database. Also, this trend could suggest that the remote islands are better studied. An increase in sampling effort and systematic studies on several islands are required to improve diversity estimates.

Recent phylogenetic studies on Mexican islands led to the description of two insular endemic rattlesnakes (Meik et al., 2018), the elevation to species of synonymous insular snake populations (Cox et al., 2018), and the proposal to elevate three subspecies of island geckos (recognized as subspecies by Ramírez‐Reyes et al., 2021; Ramírez‐Reyes & Flores‐Villela, 2018; Uetz & Hošek, 2020). Interestingly, there are no insular endemic amphibian species, despite having frog records on islands a little less than 100 km from the mainland (De La Torre et al., 2010). Due to the isolation and low migration rate, amphibian island populations likely show patterns of genetic and phenotypic divergence. There is still approximately 35% of the insular surface to be studied, mainly in the provinces of the Neotropical region. Sampling efforts and systematic studies should be prioritized, considering that at least 60% of the islands are under multiple threats, with data deficient islands likely facing a similar scenario. In Table 3, we present the ten islands with the greatest recorded threats, which can guide specific conservation efforts. Biological invasions, wildlife exploitation, and habitat modifications are the main threats to the Mexican insular herpetofauna, with Nearctic and Neotropical islands facing different situations, ultimately endangering the flora and fauna that inhabits them. The islands have and will continue to be a fundamental study model in biology, and, in the face of the biodiversity crisis, will play a leading role in the development of restoration and conservation strategies.

**TABLE 3:** The ten islands with the greatest major threats by biogeographic province and the number of amphibian and reptile species per island. The threat number indicates the data for Scenario 1 and Scenario 2, respectively

Island	Region	Province	Threat number	Species number
Isla Cozumel, QR	NT	YPP	9 (6)	38
Isla Mujeres, QR	NT	YPP	8 (5)	23
Isla Del Carmén, Cam	NT	VP	8 (5)	43
Isla Socorro, Col	NT	PLP	7 (4)	5
Isla María Madre, Nay	NT	PLP	7 (4)	22
Cayo Holbox, QR	NT	YPP	7 (4)	12
Isla San Marcos, BCS	NA	BCP	7 (4)	23
Isla San Roque, BCS	NA	BCP	7 (4)	1
Isla Grande Ixtapa, Gro	NT	BCP	6 (3)	9
Isla Roqueta, Gro	NT	PLP	6 (3)	13

## CONFLICT OF INTEREST

There are no sources, relationship, financial, or any potential sources of conflict of interest that might be perceived as influencing the objectivity of this work.

## AUTHOR CONTRIBUTION

**Juan Valentín Pliego‐Sánchez:** Data curation (equal); Formal analysis (equal); Writing‐original draft (equal); Writing‐review & editing (equal). **Christopher Balir:** Data curation (equal); Formal analysis (equal); Methodology (equal); Writing‐original draft (equal); Writing‐review & editing (equal). **Anibal Helios de la Vega Pérez:** Formal analysis (equal); Writing‐original draft (equal); Writing‐review & editing (equal). **Victor Hugo Jimenez‐Arcos:** Conceptualization (equal); Data curation (equal); Methodology (equal); Project administration (equal); Resources (equal); Supervision (equal); Writing‐original draft (equal); Writing‐review & editing (equal).

## Supporting information

Supplementary Material

## Data Availability

Data are available as appendices.
