# Supplementary material for: The insular herpetofauna of Mexico: Composition, conservation, and biogeographic patterns
Source: Ecol Evol. 2021 Apr 4;11(11):6579–92. doi: 10.1002/ece3.7513 (PMC8207341; doi:10.1002/ece3.7513)
Supplement: Supplementary file 1 — Supplementary Material [file ECE3-11-6579-s001.pdf]

The Insular Herpetofauna of Mexico: composition, conservation, and biogeographic patterns

J.V. Pliego-Sánchez, C. Blair, A.H. Díaz de la Vega-Pérez & V.H. Jiménez-Arcos

Supplementary Information contains four tables:

- Table S1. Taxonomic checklist with references
- Table S2. Island information with references
- Table S3. The 11 classes of major threats from the IUCN Red List, with associated definition obtained of Leclerc et al. (2018)
- Table S4. Threats recorded on Mexican islands

Table S1. We present the taxonomic list of the Insular Herpetofauna of Mexico with the conservation status under three classification systems and the distribution on Mexico (Endemicty level). Abbreviations: NOM059: Mexican Wildlife Conservation Standard, IUCN: The Red Book of Endangered Species, IUCN, EVS: Environmental Vulnerability Scores Index (Johnson et al., 2015; Wilson et al., 2013a, 2013b), Pr: special protection, A: threatened, P: endangered, NE: no evaluated, DD: data deficient, LC: least concern, NT: near threatened, VU: vulnerable, EN: endangered, CR: critically endangered, L: low-risk category, M: medium -risk category, H: high-risk category, ISE: endemic island species, ISB: endemic island subspecies, MX: Mexico endemic, NEM: Mexico no endemic, IAS: invasive alien species, NA: not applicable.

Taxa	Conservation status systems			Endemicity level
	NOM059	IUCN	EVS	
**AMPHIBIA (18 especies)**				
**ANURA**				
**Bufonidae (3 genera, 4 species)**				
Anaxyrus punctatus (Baird y Girard, 1852)	NL	LC	L(5)	NME
Incilius mazatlanensis (Taylor 1940)	NL	LC	M(12)	MX
Incilius valliceps (Wiegmann, 1833)	NL	LC	L(6)	NME
Rhinella horribilis (Wiegmann, 1833)	NL	LC	L(3)	NME
**Eleutherodactylidae (1 genus, 2 species)**				
Eleutherodactylus pallidus (Duellman, 1968)	Pr	DD	H(17)	MX
Eleutherodactylus planirostris (Cope, 1862)	NA	NA	NA	IAS
**Hylidae (5 genera, 5 species)**				
Dendropsophus microcephalus (Duellman 2001)	NL	LC	L(7)	NME
Hyliola regilla (Baird y Girard, 1852)	NL	LC	L(9)	NME
Scinax staufferi (Cope, 1865)	NL	LC	L(4)	NME
Smilisca baudinii (Duméril y Bibron, 1841)	NL	LC	L(3)	NME
Trachycephalus typhonius (Linnaeus, 1758)	NL	LC	L(4)	NME
**Leptodactylidae (1 genus, 1 species)**				
Leptodactylus fragilis (Brocchi, 1877)	NL	LC	L(5)	NME
Leptodactylus melanonotus (Hallowell, 1861)	NL	LC	L(6)	NME
**Microhylidae (1 genera, 1 species)**				
Hypopachus variolosus (Cope, 1866)	NL	LC	L(4)	NME
**Ranidae (1 genus, 1 species)**				
Raana berlandieri (Baird, 1859)	NL	LC	L(4)	NEM
**Scaphiopodidae (1 genus, 1 species)**				
Scaphiopus couchi (Baird, 1854)	NL	LC	L(4)	NME
**CAUDATA**				
**Plethodontidae (2 genera, 2 species)**				
Aneides lugubris (Hallowell, 1949)	Pr	LC	H(14)	NME
Batrachoseps major (Camp, 1915)	NL	LC	H(14)	NME
**REPTILIA**				
**CROCODYLIA**				
**Crocodylidae (1 genus, 2 species)**				
Crocodylus acutus (Cuvier, 1807)	Pr	VU	H(14)	NME

Crocodylus moreletii (Duméril & Bibron, 1851)	Pr	LC	M(13)	NME
**SQUAMATA**				
**AMPHISBAENIA**				
**Bipedidae (1 genus, 1 species)**				
Bipes biporus (Cope, 1894)	Pr	LC	H(14)	MX
**SAURIA**				
**Anguidae (2 genera, 5 taxa)**				
Anniella geronimensis (Shaw, 1940)	Pr	EN	M(13)	ISE
Anniella stebbinsi Papenfus & Parham 2013	Pr	LC	M(12)	MX
Elgaria cedrosensis (Fitch, 1934)	NL	LC	H(16)	ISE
Elgaria multicarinata ignava (Van Denburgh, 1905)	Pr	LC	M(10)	ISB
Elgaria multicarinata nana (Fitch, 1934)				ISB
**Corytophanidae (1 genus, 1 species)**				
Basiliscus vittatus (Wiegmann, 1828)	NL	LC	L(7)	NME
**Crotaphytidae (2 genera, 4 species)**				
Crotaphytus dickersonae (Schmidt, 1922)	NL	LC	H(16)	MX
Crotaphytus insularis (Van Denburgh & Slevin, 1921)	NL	LC	H(16)	ISE
Gambelia copeii (Yarrow, 1882)	NL	LC	M(11)	NME
Gambelia wislizenii (Baird &Girard, 1852)	Pr	LC	M(13)	NME
**Dactyloidae (1 ggenus, 6 species)**				
Anolis allisoni (Barbour, 1928)	Pr	NE	M(13)	NME
Anolis lemurinus (Cope, 1861)	NL	NE	L(8)	NME
Anolis nebulosus (Wiegmann, 1834)	NL	LC	M(13)	MX
Anolis rodriguezii (Bocourt, 1873)	NL	NE	M(10)	NME
Anolis sagrei (Duméril & Bibron, 1837)	NA	NA	NA	IAS
Anolis ustus (Cope 1864)	NL	NE	—	NME
**Eublepharidae (1 genus, 5 taxa)**				
Coleonyx elegans elegans (Gray, 1845)	A	LC	L(9)	N
Coleonyx gypsicolus (Grismer & Ottlery, 1988)	NL	LC	H(18)	IS-E
Coleonyx variegatus abbotti Klauber, 1945				
Coleonyx variegatus sonoriensis Klauber, 1945	Pr	LC	M(11)	NME
Coleonyx variegatus variegatus (Baird, 1858)				
**Gekkonidae (2 genera, 3 species)**				
Gehyra mutilata (Wiegmann, 1834)	NA	NA	NA	IAS
Hemidactylus frenatus (Duméril & Bibron, 1836)	NA	NA	NA	IAS
Hemidactylus turcicus (Linnaeus, 1758)	NA	NA	NA	IAS
**Iguanidae (4 genera, 15 species)**				
Ctenosaura acanthura (Shaw 1802)	Pr			
Ctenosaura conspicuosa (Dickerson, 1919)	NL	VU	H(16)	ISE

Ctenosaura hemilopha (Cope, 1863)	Pr	NE	H(18)	MX
Ctenosaura nolascensis (Smith, 1972)	NL	VU	H(17)	ISE
Ctenosaura pectinata (Wiegmann, 1834)	A	NE	H(15)	MX
Ctenosaura similis (Gray, 1831)	A	LC	L(8)	NME
Dipsosaurus catalinensis (Van Denburgh, 1922)	NL	NE	H(17)	ISE
Dipsosaurus dorsalis dorsalis (Baird & Girard 1852)	NL	LC	M(11)	NME
Iguana iguana (Linnaeus, 1758)	Pr	LC	M(12)	NME
Sauromalus ater ater Duméril, 1856<br>*Sauromalus ater towsendi* Dickerson, 1919	Pr	LC	M(13)	NME
Sauromalus hispidus (Stejneger, 1891)	A	EN	H(14)	ISE
Sauromalus klauberi (Shaw, 1941)	P	VU	H(16)	ISE
Sauromalus sleveni (Van Denburgh, 1922)	A	NT	H(16)	ISE
Sauromalus varius (Dickerson, 1919)	A	VU	H(16)	ISE
**Mabuyidae (1 genus, 1 species)**				
Marisora brachypoda (Taylor, 1956)	NL	LC	L(6)	NME
**Prhynosomatidae (6 genera, 40 taxa)**				
Callisaurus draconoides brevipes Bogert & Dorson 1942				MX
Callisaurus draconoides carmensis Dickerson 1919				MX
Callisaurus draconoides draconoides Blainville 1835	A			NME
Callisaurus draconoides inusitatus Dickerson 1919				MX
Callisaurus draconoides rhodostictus Cope, 1896				NME
Petrosaurus mearnsi (Stejneger, 1894)	Pr	LC	M(12)	NME
Petrosaurus repens (Van Denburgh, 1895)	NL	LC	M(13)	MX
Petrosaurus slevini (Van Denburgh, 1922)	NL	LC	H(16)	ISE
Petrosaurus thalassinus (Cope, 1863)	Pr	LC	M(13)	MX
Phrynosoma cerroense (Stejneger, 1893)	A	NE	H(16)	ISE
Phrynosoma solare (Gray, 1845)	NL	LC	M(14)	NME
Sceloporus angustus (Dickerson, 1919)	A	LC	H(16)	ISE
Sceloporus chrysostictus (Cope, 1867)	NL	LC	M(13)	NME
Sceloporus clarkii clarkii (Baird & Girard, 1852)	NL	LC	M(10)	NME
Sceloporus cozumelae (Jones, 1927)	Pr	LC	H(15)	MX
Sceloporus grandaevus (Dickerson, 1919)	A	LC	H(16)	ISE
Sceloporus hunsakeri (Hall & Smith, 1979)	A	LC	H(14)	MX
Sceloporus lineatulus (Dickerson, 1919)	A	LC	H(17)	ISE
Sceloporus magister magister (Hallowell, 1854)	NL	LC	L(9)	NME
Sceloporus occidentalis occidentalis (Baird & Girard, 1852)	NL	LC	M(12)	NME
Sceloporus orcutti (Stejneger, 1893)	NL	LC	L(7)	NME
Sceloporus variabilis variabilis (Cope, 1869)	NL	LC	L(5)	NME
Sceloporus zosteromus monserratensis Van Denburgh & Slevin, 1920	Pr	LC	M(12)	MX

Sceloporus zosteromus rufidorsum Yarrow 1881	Pr	LC	M(12)	MX
Urosaurus auriculatus (Cope, 1871)	Pr	LC	M(12)	ISE
Urosaurus bicarinatus bicarinatus (Duméril, 1856)	NL	LC	M(12)	MX
Urosaurus bicarinatus tuberculatus (Duméril, 1856)				
Urosaurus clarionensis (Townsend, 1890)	NL	VU	H(17)	ISE
Urosaurus nigricaudus (Cope, 1864)	A	LC	L(8)	NME
Urosaurus ornatus schottii (Baird & Girard, 1852)	—	LC	M(10)	MX
Uta encantadae (Grismer, 1994)	NL	VU	H(17)	ISE
Uta lowei (Grismer, 1994)	NL	VU	H(17)	ISE
Uta nolascensis (Van Denburgh & Slevin, 1921)	A	LC	H(17)	ISE
Uta palmeri (Stejneger, 1890)	A	VU	H(17)	ISE
Uta squamata (Dickerson, 1919)	A	LC	H(17)	ISE
Uta stansburiana elegans (Baird & Girard, 1852)	A	LC	L(7)	NME
Uta stansburiana martinensis (Baird & Girard, 1852)				ISB
Uta stansburiana stejnegeri (Baird & Girard, 1852)				NME
Uta stansburiana taylori (Baird & Girard, 1852)				NME
Uta tumidarostra (Grismer, 1994)	NL	VU	H(17)	ISE
**Phyllodactylidae (1 genus, 13 taxa)**				
Phyllodactylus bugastrolepis (Dixon, 1966)	A	LC	H(17)	ISE
Phyllodactylus homolepidurus homolepidurus Smith 1935	Pr	LC	H(15)	MX
Phyllodactylus homolepidurus nolascoensis Dixon 1964				ISB
Phyllodactylus lanei isabelae Castro-Franco & Uribe-Peña 1992	NL	LC	H(15)	ISB
Phyllodactylus lanei lanei Smith 1935				MX
Phyllodactylus lanei lupitae Castro-Franco & Uribe-Peña 1992				ISB
Phyllodactylus lanei rupinus Dixon 1964				MX
Phyllodactylus nocticolus Dixon, 1964	Pr	LC	M(10)	NEM
Phyllodactylus partidus Dixon, 1966	Pr	LC	H(16)	ISE
Phyllodactylus tuberculosus saxatilis Dixon 1964	NL	LC	L(8)	NEM
Phyllodactylus unctus (Cope, 1863)	Pr	NT	H(15)	MX
Phyllodactylus xanti xanti Cope 1863	Pr	LC	H(15)	MX
Phyllodactylus xanti zweifeli Dixon, 1964				MX
**Scincidae (2 genera, 4 taxa)**				
Mesoscincus schwartzei (Fischer, 1884)	NL	LC	M(11)	NEM
Plestiodon gilberti rubricaudatus (Taylor 1936)	Pr	LC	M(12)	NEM
Plestiodon skiltonianus interparietalis (Tanner 1958)	NL	LC	M(11)	NEM
**Sphaerodactylidae (2 genera, 3 species)**				
Aristelliger georgeensis (Bocourt, 1873)	Pr	LC	M(13)	NEM
Sphaerodactylus continentalis Werner, 1896	NL	NE	M(10)	NEM
Sphaerodactylus glaucus Cope, 1866	Pr	LC	M(12)	NEM

**Teiidae (2 genera, 29 taxa)**				
Aspidoscelis carmenensis (Maslin & Secoy, 1986)	NL	LC	H(17)	ISE
Aspidoscelis ceralbensis (Van Denburgh & Slevin, 1921)	Pr	LC	H(17)	ISE
Aspidoscelis communis mariarum (Günther, 1885)	Pr	LC	H(14)	MX
Aspidoscelis costatus huico (Zweifel, 1959)	Pr	LC	M(11)	MX
Aspidoscelis costatus nigrigularis (Zweifel, 1959)				MX
Aspidoscelis cozumelae (Gadow, 1906)	A	LC	H(16)	ISE
Aspidoscelis danheimae (Burt, 1929)	A	LC	H(16)	ISE
Aspidoscelis deppii deppii (Wiegmann, 1834)	NL	LC	L(8)	NEM
Aspidoscelis espiritensis (Van Denburgh & Slevin, 1921)	A	LC	H(16)	ISE
Aspidoscelis franciscensis (Van Denburgh & Slevin, 1921)	NL	LC	H(17)	ISE
Aspidoscelis gularis gularis (Baird & Girard 1852)	NL	LC	L(9)	NEM
Aspidoscelis guttatus guttatus (Wiegmann 1834)	NL	LC	M(12)	MX
Aspidoscelis guttatus immutabilis (Cope 1878)				MX
Aspidoscelis hyperythrus hyperythus (Cope 1863)	A	LC	M(10)	NEM
Aspidoscelis hyperythrus schmidti (Van Denburgh & Slevin 1921)	Pr			NEM
Aspidoscelis lineattissimus lineattissimus (Cope 1878)	A	LC	H(14)	MX
Aspidoscelis lineattissimus duodecemlineatus (Lewis 1956)				MX
Aspidoscelis martyris (Stejneger, 1891)	NL	LC	H(17)	ISE
Aspidoscelis maslini (Fritts, 1969)	A	LC	H(15)	NEM
Aspidoscelis pictus (Van Denburgh & Slevin, 1921)	A	LC	H(17)	ISE
Aspidoscelis rodecki (McCoy & Maslin, 1962)	P	NT	H(16)	MX
Aspidoscelis tigris aethiops (Cope 1900)				MX
Aspidoscelis tigris dickersonae (Van Denburgh & Slevin 1921)				ISB
Aspidoscelis tigris multiscutatus (Cope 1892)	NL	LC	L(8)	NEM
Aspidoscelis tigris rubidus (Cope 1892)				MX
Aspidoscelis tigris vividus (Walker 1891)				NEM
Holcosus undulatus amphigrammus (Smith & Laufe 1945)				MX
Holcosus undulatus gaigae (Smith & Laufe 1946)	NL	NE	L(7)	MX
Holcosus undulatus stuarti (Smith 1940)				MX
**SERPENTES**				
**Boidae (2 genera, 2 species)**				
Boa imperator (Daudin, 1803)	A	LC	NL	NEM
Lichanura trivirgata (Cope, 1861)	A	LC	M(10)	NEM
**Colubridae (Colubrinae; 17 genera, 44 taxa)**				
Bogertophis rosaliae (Mocquard, 1899)	NL	LC	M(10)	NEM
Drymachon melanurus (Duméril, Bibron & Duméril, 1854)	NL	LC	L(6)	NEM
Drymobius margaritiferus margaritiferus (Schlegel 1837)	NL	LC	L(6)	NEM
Lampropeltis abnorma (Bocourt, 1886)	NL	LC	—	NEM

Lampropeltis catalinensis (Van Denburgh & Slevin, 1921)	NL	DD	H(17)	ISE
Lampropeltis californiae (Blainville, 1835)	A	LC	M(10)	NEM
Lampropeltis polyzona Cope, 1860	A	LC	M(11)	MX
Lampropeltis zonata (Lockington, 1835)	A	LC	H(15)	NEM
Leptophis diplotropis forreri Smith 1943	A	LC	H(14)	ISB
Leptophis mexicanus yucatanensis Oliver 1942	A	LC	L(6)	NEM
Masticophis anthonyi (Stejneger, 1901)	A	CR	H(17)	ISE
Masticophis barbouri (Van Denburgh & Slevin, 1921)	A	DD	H(17)	ISE
Masticophis bilineatus (Jan, 1863)	NL	LC	M(11)	NEM
Masticophis flagellum cingulum Lowe & Woodin 1954	A	LC	L(8)	NEM
Masticophis fuliginosus (Cope, 1895)	NL	NE	L(9)	NEM
Masticophis mentovarius variolosus (Smith, 1943)	A	LC	L(6)	ISB
Masticophis slevini Lowe & Norris, 1955	NL	LC	H(17)	ISE
Mastigodryas melanolomus melanolomus (Cope 1868)	NL	LC	L(6)	NEM
Mastigodryas melanolomus slevini (Stuart 1933)				ISB
Oxybelis aeneus (Wagler, 1824)	NL	LC	L(5)	NEM
Oxybelis fulgidus (Daudin, 1803)	NL	LC	L(9)	NEM
Phyllorhynchus decurtatus (Cope, 1868)	NL	LC	M(11)	NEM
Pituophis catenifer afinis Hallowell 1852	NL	LC	L(9)	NEM
Pituophis catenifer fulginatus Klauber 1946				NEM
Pituophis insulanus (Klauber, 1946)	NL	LC	H(16)	ISE
Pituophis vertebralis (Blainville, 1835)	NL	LC	M(12)	MX
Pseudoelaphe flavirufa flavirufa (Cope 1866)	Pr	LC	M(10)	NEM
Rhinocheilus etheridgei Grismer, 1990	A	DD	H(16)	IS-E
Salvadora hexalepis hexalepis Cope, 1867	NL	LC	M(10)	NEM
Salvadora hexalepis virgultea Bogert, 1936				NEM
Salvadora mexicana (Duméril, Bibron & Duméril, 1854)	Pr	LC	H(15)	MX
Spillotes pullatus (Linnaeus, 1758)	NL	LC	L(6)	NEM
Sonora cincta (Cope, 1861)	Pr	NE	H (14)	NEM
Sonora fasciata (Cope 1892)	NL	NE	H (18)	MX
Sonora mosaueri Stickel 1938	NL	NE	H (16)	MX
Sonora punctatissima Van Denburgh & Slevin, 1921	Pr	NE	M (13)	ISE
Sonora savagei (Cliff, 1954)	Pr	LC	H(15)	ISE
Tantilla bocourti (Günther, 1895)	NL	LC	L(9)	MX
Tantilla calamarina Cope, 1866	Pr	LC	M(12)	MX
Tantilla moesta (Günther, 1863)	NL	LC	M(13)	NEM
Tantilla planiceps (Blainville, 1835)	NL	LC	L(9)	NEM
Trimorphodon biscutatus (Cope, 1860)	NL	LC	L(7)	NEM
Trimorphodon lambda (Cope, 1860)	NL	LC	M(13)	NEM

<table>
<tr><td>Trimorphodon lyrophanes (Cope, 1860)</td><td>NL</td><td>LC</td><td>M(10)</td><td>NEM</td></tr>
<tr><td colspan="5">Colubridae (Dipsadinae; 12 genera, 26 taxa)</td></tr>
<tr><td>Coniophanes imperialis imperialis (Baird 1859)</td><td>NL</td><td>LC</td><td>L(8)</td><td>NEM</td></tr>
<tr><td>Conophis lineatus (Duméril, Bibron & Duméril, 1854)</td><td>NL</td><td>LC</td><td>L(9)</td><td>NEM</td></tr>
<tr><td>Diadophis punctatus (Linnaeus, 1766)</td><td>NL</td><td>LC</td><td>L(4)</td><td>NEM</td></tr>
<tr><td>Dipsas brevifacies (Cope, 1866)</td><td>Pr</td><td>LC</td><td>H(15)</td><td>NEM</td></tr>
<tr><td>Hypsiglena catalinae (Tanner 1966)</td><td>Pr</td><td>NE</td><td>NL</td><td>ISE</td></tr>
<tr><td>Hypsiglena chlorophaea tiburonensis Tanner 1981</td><td>Pr</td><td>LC</td><td>L(8)</td><td>ISB</td></tr>
<tr><td>Hypsiglena ochrorhynchus baueri (Zweifel 1958)</td><td rowspan="6">Pr</td><td rowspan="6">LC</td><td rowspan="6">L(8)</td><td>ISB</td></tr>
<tr><td>Hypsiglena ochrorhynchus gularis Cope 1860</td><td>ISB</td></tr>
<tr><td>Hypsiglena ochrorhynchus klauberi Tanner 1946</td><td>NEM</td></tr>
<tr><td>Hypsiglena ochrorhynchus martinensis (Tanner & Banta 1962)</td><td>ISB</td></tr>
<tr><td>Hypsiglena ochrorhynchus ochorhyncus (Cope 1860)</td><td>MX</td></tr>
<tr><td>Hypsiglena ochrorhynchus venusta (Mocquard 1899)</td><td>MX</td></tr>
<tr><td>Hypsiglena slevini marcoensis Ottley & Tanner 1978</td><td rowspan="2">A</td><td>LC</td><td>L(6)</td><td>ISB</td></tr>
<tr><td>Hypsiglena slevini slevini Tanner 1943</td><td>LC</td><td>M(12)</td><td>MX</td></tr>
<tr><td>Hypsiglena torquata (Günther, 1860)</td><td>Pr</td><td>LC</td><td>L(8)</td><td>NEM</td></tr>
<tr><td>Hypsiglena unaocularus (Tanner, 1946)</td><td>Pr</td><td>NE</td><td>NL</td><td>ISE</td></tr>
<tr><td>Imantodes cenchoa (Linnaeus, 1758)</td><td>Pr</td><td>LC</td><td>L(6)</td><td>NEM</td></tr>
<tr><td>Imantodes gemmistratus latistratus (Cope 1887)</td><td>Pr</td><td>LC</td><td>L(6)</td><td>MX</td></tr>
<tr><td>Leptodeira frenata malleisi Dunn & Stuart 1935</td><td rowspan="2">NL</td><td rowspan="2">LC</td><td rowspan="2">M(12)</td><td>NEM</td></tr>
<tr><td>Leptodeira frenata yucatanensis (Cope 1887)</td><td>MX</td></tr>
<tr><td>Pseudoleptodeira latifasciata (Günther, 1894)</td><td>Pr</td><td>LC</td><td>H(14)</td><td>MX</td></tr>
<tr><td>Ninia sebae sebae (Duméril, Bibron & Duméril 1854)</td><td>NL</td><td>LC</td><td>L(5)</td><td>NEM</td></tr>
<tr><td>Rhadinaea hesperia (Bailey, 1940)</td><td>NL</td><td>LC</td><td>M(10)</td><td>MX</td></tr>
<tr><td>Sibon nebulatus (Linnaeus, 1758)</td><td>NL</td><td>LC</td><td>L(5)</td><td>NEM</td></tr>
<tr><td>Tropidodipsas annulifera (Boulenger, 1894)</td><td>Pr</td><td>LC</td><td>M(13)</td><td>MX</td></tr>
<tr><td>Tropidodipsas sartorii (Cope, 1863)</td><td>Pr</td><td>LC</td><td>L(9)</td><td>NEM</td></tr>
<tr><td colspan="5">Elapidae (Hydrophiinae; 1 genus, 1 species)</td></tr>
<tr><td>Hydrophis platurus platurus (Linneaus, 1766)</td><td>NL</td><td>LC</td><td>NL</td><td>NEM</td></tr>
<tr><td colspan="5">Elapidae (Colubroidea; 2 genera, 2 species)</td></tr>
<tr><td>Micruroides euryxanthus euryxanthus (Kennicott, 1860)</td><td>A</td><td>LC</td><td>H(15)</td><td>NEM</td></tr>
<tr><td>Micrurus diastema sapperi (Werner 1903)</td><td>Pr</td><td>LC</td><td>L(8)</td><td>NEM</td></tr>
<tr><td colspan="5">Leptotyphlopidae (2 genera, 2 species)</td></tr>
<tr><td>Epictia magnamaculata Taylor, 1940</td><td>NL</td><td>LC</td><td>NL</td><td>NEM</td></tr>
<tr><td>Rena humilis humilis Baird & Girard, 1853</td><td>NL</td><td>LC</td><td>L(8)</td><td>NEM</td></tr>
<tr><td colspan="5">Natricidae (2 genera, 2 species)</td></tr>
<tr><td>Nerodia rhombifer werleri (Conant 1953)</td><td>NL</td><td>LC</td><td>M(10)</td><td>MX</td></tr>
<tr><td>Thamnophis proximus proximus (Say 1823)</td><td>A</td><td>LC</td><td>L(7)</td><td>NEM</td></tr>
</table>

**Typhlopidae (1 genus, 1 species)**				
Indotyphlops braminus (Daudin, 1803)	NA	NA	NA	IAS
**Viperidae (2 genera, 19 taxa)**				
Agkistrodon bilineatus Günther, 1863	Pr	NT	M(11)	NEM
Crotalus angelensis Klauber, 1963	NL	LC	H(18)	ISE
Crotalus atrox Baird & Girard, 1853	Pr	LC	L(9)	NEM
Crotalus basiliscus (Cope, 1864)	Pr	LC	H(16)	MX
Crotalus catalinensis Cliff, 1954	A	CR	H(19)	ISE
Crotalus cerastes cerastes Cliff 1954	Pr	LC	H(16)	NEM
Crotalus enyo cerralvensis (Cope,1861)	—	LC	H(19)	ISB
Crotalus enyo enyo (Cope, 1861)				MX
Crotalus estebanensis Klauber, 1949	—	LC	H(19)	ISE
Crotalus helleri Meek, 1905	Pr	LC	M(12)	NEM
Crotalus lorenzoensis Radcliff & Maslin, 1975	Pr	LC	L(8)	ISE
Crotalus mitchellii (Cope, 1861)	—	NE	—	MX
Crotalus molossus nigrescens Gloyd 1936	—	NE	—	NEM
Crotalus polisi Meik, Schaack, Flores-Villela & Streicher, 2018	Pr	LC	L(9)	ISE
Crotalus pyrrhus (Cope, 1866)	—	NE	—	NEM
Crotalus ruber exsul Cope, 1892	Pr	LC	H(16)	NEM
Crotalus ruber lucasensis Van Denburg 1920				ISE
Crotalus thalassoporus Meik, Schaack, Flores-Villela & Streicher, 2018	NL	NE	H(19)	ISE
Crotalus tigris Kennicott, 1859	Pr	LC	H(16)	NEM
**TESTUDINES**				
**Cheloniidae (4 genera, 6 taxa)**				
Caretta caretta (Linnaeus, 1758)	P	VU	NL	NEM
Chelonia mydas (Linnaeus, 1758)	P	EN	NL	NEM
Eretmochelys imbricata bissa (Rüppell 1835)	P	CR	NL	NEM
Eretmochelys imbricata imbricat (Linnaeus 1766)				NEM
Lepidochelys kempii Garman, 1880	P	CR	NL	NEM
Lepidochelys olivacea (Eschscholtz, 1829)	P	VU	NL	NEM
**Dermatemydidae (1 genus, 1 species)**				
Dermatemys mawii Gray, 1847	P	CR	H(17)	NEM
**Dermochelydae (1 genus, 1 species)**				
Dermochelys coriacea (Vandelli, 1761)	P	CR	NL	NEM
**Emydidae (1 genus, 1 species)**				
Trachemys venusta (Gray, 1855)	NL	NE	M(13)	NEM
**Geoemydidae (1 genus, 1 species)**				
Rhinoclemmys areolata (Duméril, Bibron & Duméril, 1851)	A	NT	M(13)	NEM
**Kinosternidae (2 genera, 4 taxa)**				

Kinosternon herrerai Stejneger, 1925	Pr	NT	H(14)	MX
Kinosternon integrum (Le Conte, 1854)	Pr	LC	M(11)	MX
Kinosternon scorpioides cruentatum Duméril, Bibron & Duméril 1851	Pr	NE	M(10)	NEM
Staurotypus triporcatus (Wiegmann, 1828)	A	NT	H(14)	NEM
**Testudinidae (1 genus, 1 species)**				
Gopherus morafkai Murphy, Berry, Edwards, Levinton, Lathrop & Riedle, 2011	NL	NE	H(15)	NEM

Table S2. The information collected of area, mainland distance (MD), species numbers for amphibians, reptiles, herpetofauna, and sources (data obtained from the collection review; DCR) are shown.

Island Name	Area (Km2)	MD (km)	Amphibians	Reptiles	Herpetofauna	References
Cayo Arcas, Cam	0.210	144.840	0	1	1	(González-Sánchez et al. 2017)
Cayo Centro, QR	5.300	42.330	0	10	10	(Charruau et al. 2015)
Cayo Contoy, QR	2.151	6.000	0	15	15	(González-Sánchez et al. 2017)
Cayo Culebra, QR	0.710	8.450	0	1	1	(González-Sánchez et al. 2017)
Cayo de Enmedio, Ver	0.037	5.970	0	1	1	DCR
Cayo Holbox, QR	52.131	0.050	1	11	12	(González-Sánchez et al. 2017)
Cayo Isla Verde, Ver	0.029	5.940	0	2	2	DCR
Cayo Lobos, QR	0.003	41.730	0	2	2	(Charruau et al. 2015)
Cayo Lobos, Ver	0.180	12.110	0	2	2	DCR
Cayo Norte, QR	0.340	38.600	0	11	11	(Charruau et al. 2015)
Cayo Pérez, Yuc	0.111	104.607	1	6	7	DCR
Isla Alcatraz, Son	0.450	1.56	0	3	3	(Grismer 2002)
Isla Ángel de la Guarda, BC	936.040	12.12	0	15	15	(Grismer 2002)
Isla Asunción, BCS	0.430	1.47	0	1	1	(Grismer 2002; Samaniego-Herrera et al. 2007)
Isla Ballena, BCS	0.460	28.50	0	6	6	(Grismer 2002)
Isla Bota, BC	0.100	0.73	0	1	1	(Grismer 2002)
Isla Brosa, BCS	9.000	3.15	0	1	1	(Grismer 2002)
Isla Cabeza de Caballo, BC	0.770	2.00	0	3	3	(Grismer 2002)
Isla Cardonosa Este, BC	0.140	19.30	0	3	3	(Grismer 2002)
Isla Cayo, BCS	0.020	1.58	0	2	2	(Grismer 2002)
Isla Cedros, BC	348.300	22.75	1	14	15	(Grismer 2002; Samaniego-Herrera et al. 2007)
Isla Cerraja, BC	0.030	3.00	0	1	1	(Grismer 2002)
Isla Cerralvo, BCS	40.460	12.10	2	19	21	(Grismer 2002)
Isla Chapetona, Son	0.100	0.73	0	1	1	DCR
Isla Clarión, Col	19.577	700.000	0	5	5	(Mulcahy et al., 2014)/DCR
Isla Cocinas, Jal	0.355	1.850	0	6	6	(Hernández-Salinas et al. 2014)/DCR
Isla Coloradito, BC	0.190	7.71	0	1	1	(Grismer 2002)
Isla Coronado Medio, BC	0.118	14.40	1	2	3	(Grismer 2002; Samaniego-Herrera et al. 2007)
Isla Coronado Norte, BC	0.390	18.00	2	7	9	(Grismer 2002; Samaniego-Herrera et al. 2007)
Isla Coronado Sur, BC	1.222	13.00	1	8	9	(Grismer 2002; Samaniego-Herrera et al. 2007)
Isla Coronados, BCS	7.180	2.86	0	16	16	(Grismer 2002; Arnaud & Blázquez 2018)
Isla Coyote, BCS	0.250	0.81	0	3	3	(Grismer 2002)
Isla Cozumel, QR	467.889	18.000	8	30	38	(González-Sánchez et al. 2017)
Isla Danzante, BCS	4.640	2.61	0	16	16	(Grismer 2002)
Isla de Burros, Ver	0.728	3.290	0	1	1	DCR
Isla del Carmen, BCS	142.360	7.00	0	16	16	(Grismer 2002)
Isla Del Carmén, Cam	142.360	3.290	6	37	43	(González-Sánchez et al. 2017; DCR)
Isla El Borrego, BC	0.100	2.88	0	2	2	(Grismer 2002)
Isla El Farallón, Sin	0.160	25.000	0	1	1	(Peralta-García et al., 2007)
Isla El Muerto, BCS	1.000	3.39	0	5	5	(Grismer 2002)
Isla El Pardito, BCS	0.100	9.35	0	2	2	(Grismer 2002)
Isla El Pelícano, Son	18.060	4.00	0	1	1	DCR
Isla El Requeson, BCS	0.140	0.20	0	1	1	(Grismer 2002)
Isla El Toro, Ver	3.293	7.000	3	6	9	DCR
Isla Encantada, BC	0.440	7.00	0	1	1	(Grismer 2002)
Isla Espiritu Santo, BCS	83.790	6.40	2	19	21	(Grismer 2002; Lindell et al. 2005)
Isla Estanque, BC	0.760	31.85	0	5	5	(Grismer 2002)
Isla Gallina, BCS	0.017	30.40	0	2	2	(Grismer 2002)
Isla Gallo, BCS	0.056	30.00	0	5	5	(Grismer 2002)
Isla Gaviota, BCS	0.140	0.50	0	1	1	(Grismer 2002)
Isla Grande Ixtapa, Gro	0.333	0.850	0	9	9	(Gaviño-de la Torre et al. 1979)/DCR
Isla Granito, BC	0.190	29.57	0	2	2	(Grismer 2002)
Isla Isabela, Nay	1.940	29.20	0	8	8	(Casas-Andreu 1992)
Isla Islitas, BCS	0.019	0.44	0	1	1	(Grismer 2002)
Isla Juana Ramírez, Ver	27.678	0.370	0	1	1	DCR
Isla La Larga, Nay	0.344	7.81	0	9	9	(Casas-Andreu 1992)
Isla La Peña, Nay	0.120	1.78	1	8	9	(Ramírez-Reyes et al. 2015)
Isla La Rasa, BC	0.530	20.00	0	2	2	(Grismer 2002)
Isla La Redonda, Nay	0.346	6.61	0	9	9	(Casas-Andreu 1992)
Isla La Ventana, BC	1.140	3.17	0	3	3	(Grismer 2002)
Isla Lagartija, BC	0.020	18.96	0	1	1	(Grismer 2002)
Isla Las Ánimas, BCS	0.090	27.00	0	2	2	(Grismer 2002)
Isla Las Galeras Este, BCS	0.050	17.40	0	1	1	(Grismer 2002)
Isla Las Galeras Oeste, BCS	0.030	17.00	0	1	1	(Grismer 2002)
Isla Los Patos, Sin	0.156	1.59	0	1	1	DCR
Isla Magdalena, BCS	287.110	1.33	0	14	14	(Grismer 2002; Peralta-García et al., 2007; Samaniego-Herrera et al. 2007)
Isla María Cleofas, Nay	25.000	87.50	2	11	13	(Casas-Andreu 1992)
Isla María Madre, Nay	145.300	99.50	4	18	22	(Casas-Andreu 1992; de la Torre et al. 2010)
Isla María Magdalena, Nay	70.400	97.00	1	16	17	(Casas-Andreu 1992)
Isla Mejia, BC	2.440	24.65	0	6	6	(Grismer 2002)
Isla Mitlán, BCS	0.140	2.18	0	2	2	(Grismer 2002)
Isla Monserrat, BCS	18.440	15.49	0	13	13	(Grismer 2002)
Isla Mosca, BCS	0.036	0.42	0	1	1	(Grismer 2002)
Isla Mujeres, QR	3.857	5.570	1	22	23	(González-Sánchez et al. 2017)
Isla Natividad, BCS	7.280	9.30	0	2	2	(Grismer 2002; Samaniego-Herrera et al. 2007)
Isla Pájaros, Sin	0.542	0.92	0	1	1	DCR
Isla Pardo, BCS	0.029	0.42	0	4	4	(Grismer 2002)
Isla Partida Norte, BC	0.790	18.18	0	3	3	(Grismer 2002)
Isla Partida, BCS	17.717	29.72	2	18	20	(Grismer 2002)
Isla Pata, BCS	3.410	0.75	0	1	1	(Grismer 2002)
Isla Pata, BCS	0.110	4.18	0	1	1	(Grismer 2002)
Isla Patos, Son	0.340	9.06	0	2	2	(Grismer 2002)
Isla Pérez, Ver	0.660	1.330	1	0	1	DCR
Isla Piedra, BCS	0.570	2.46	0	1	1	(Grismer 2002)

Isla Piedra, Cam	0.100	0.050	1	6	7	DCR
Isla Piojo, BC	0.570	8.50	0	4	4	(Grismer 2002)
Isla Roca Lobos, BC	0.015	20.54	0	2	2	(Grismer 2002)
Isla Roqueta, Gro	0.754	0.300	0	13	13	(González-Capistran 1986)/This study
Isla Salsipuedes, BC	0.970	19.500	0	6	6	(Grismer 2002)
Isla San Benito Este, BC	1.420	140.00	0	1	1	(Grismer 2002; Samaniego-Herrera et al. 2007)
Isla San Benito Medio, BC	0.430	142.75	0	1	1	(Grismer 2002; Samaniego-Herrera et al. 2007)
Isla San Benito Oeste, BC	3.860	145.50	0	1	1	(Grismer 2002; Samaniego-Herrera et al. 2007)
Isla San Cosme, BCS	0.340	1.46	0	3	3	(Grismer 2002)
Isla San Damian, BCS	0.045	2.58	0	1	1	(Grismer 2002)
Isla San Diego, BCS	0.520	19.23	0	3	3	(Grismer 2002)
Isla San Esteban, Son	40.000	16.57	0	9	9	(Grismer 2002)
Isla San Francisco, BCS	3.780	7.50	0	10	10	(Grismer 2002)
Isla San Ignacio, Sin	36.700	2.03	0	3	3	DCR
Isla San Ildefonso, BCS	1.330	9.94	0	4	4	(Grismer 2002)
Isla San Jerónimo, BC	0.483	9.70	0	3	3	(Grismer 2002; Samaniego-Herrera et al. 2007)
Isla San José, BCS	180.790	5.49	0	22	22	(Grismer 2002)
Isla San Juanito, Nay	9.100	108.50	0	6	6	(Casas-Andreu 1992)
Isla San Lorenzo Norte, BC	3.995	20.60	0	5	5	(Grismer 2002)
Isla San Lorenzo, BC	32.058	16.55	0	7	7	(Grismer 2002)
Isla San Luis, BC	6.067	6.32	0	4	4	(Grismer 2002)
Isla San Marcos, BCS	28.669	5.24	0	23	23	(Grismer 2002)
Isla San Martín, BC	2.560	5.10	0	6	6	(Grismer 2002; Samaniego-Herrera et al. 2007)
Isla San Pancho, Jal	0.040	1.020	0	6	6	(Hernández-Salinas et al. 2014)/DCR
Isla San Pedro Martir, BC	2.729	51.00	0	4	4	(Grismer 2002)
Isla San Pedro Nolasco, Son	3.482	19.43	0	6	6	(Grismer 2002)
Isla San Roque, BCS	0.370	1.90	0	1	1	(Grismer 2002; Samaniego-Herrera et al. 2007)
Isla Santa Catalina, BCS	38.897	25.15	0	11	11	(Grismer 2002)
Isla Santa Cruz, BCS	13.042	20.38	0	6	6	(Grismer 2002)
Isla Santa Inez, BCS	0.280	5.01	0	2	2	(Grismer 2002)
Isla Santa Margarita, BCS	217.610	3.50	1	16	17	(Grismer 2002; Peralta-García et al., 2007; Samaniego-Herrera et al. 2007)
Isla Santa María, Sin	25.882	0.36	0	1	1	DCR
Isla Santiago, BCS	0.033	1.84	0	1	1	(Grismer 2002)
Isla Smith, BC	8.032	2.99	0	7	7	(Grismer 2002)
Isla Socorro, Col	131.321	576.500	0	5	5	DCR
Isla Techal, QR	3.530	1.840	0	1	1	(Jiménez-Arcos et al. 2020)
Isla Tiburón, Son	1198.748	2.72	2	26	28	(Grismer 2002)
Isla Tijeras, BCS	0.023	1.96	0	1	1	(Grismer 2002)
Isla Todos Santos Norte, BC	0.307	18.30	0	2	2	(Grismer 2002; Peralta-García et al., 2007; Samaniego-Herrera et al. 2007)
Isla Todos Santos Sur, BC	0.870	17.30	1	7	8	(Grismer 2002; Samaniego-Herrera et al. 2007)
Isla Tortuga, BC	11.302	35.90	0	4	4	(Grismer 2002)
Isla Turners, Son	1.280	29.50	0	4	4	(Grismer 2002)
Isla Venados, Sin	0.562	1.23	0	1	1	DCR
Isla Willard, BC	1.500	0.03	0	1	1	(Grismer 2002)
Islote Peña Blanca, Col	0.110	1.830	0	1	1	DCR
Islotes Blancos, BC	0.010	7.93	0	1	1	(Grismer 2002)
Roca Cholludo, Son	0.004	30.00	0	2	2	(Grismer 2002)

Table S3. The 11 major threats from the IUCN Red List, with associated definition. We also present the original threat names from Salafsky et al. (2008) in parenthesis according to Leclerc et al. (2018) on which we are based.

**IUCN - CMP Unified Classification of Direct Threats**	
1. Cultivation<br>(agriculture & aquaculture)	Threats from farming and ranching as a result of agricultural expansion and intensification, including silviculture, mariculture and aquaculture (includes the impacts of any fencing around farmed areas).
2. Wildlife exploitation<br>(biological resource use)	Threats from consumptive use of "wild" biological resources including both deliberate and unintentional harvesting effects; also, persecution or control of specific species.
3. Climate change<br>(climate change & severe weather)	Threats from long-term climatic changes, which may be linked to global warming and other severe climatic/weather events that are outside of the natural range of variation, or potentially can wipe out a vulnerable species or habitat.
4. Energy production & Mining	Threats from production of non-biological resources.
5. Geological events	Threats from catastrophic geological events.
6. Human intrusions & disturbance	Threats from human activities that alter, destroy and disturb habitats and species associated with non-consumptive uses of biological resources.
7. Biological Invasions<br>(invasive & other problematic species, genes & diseases)	Threats from non-native and native plants, animals, pathogens/microbes, or genetic materials that have or are predicted to have harmful effects on biodiversity following their introduction, spread and/or increase in abundance.
8. Habitat modifications<br>(natural system modifications)	Threats from actions that convert or degrade habitat in service of "managing" natural or semi-natural systems, often to improve human welfare.
9. Pollution	Threats from introduction of exotic and/or excess materials or energy from point and nonpoint sources.
10. Urbanization<br>(residential & commercial development)	Threats from human settlements or other non-agricultural land uses with a substantial footprint.
11. Transport corridors<br>(transportation & service corridors)	Threats from long narrow transport corridors and the vehicles that use them including associated wildlife mortality.

References

Leclerc C, Courchamp F, Bellard C (2018) Insular threat associations within taxa worldwide. Sci Rep 8:1–8. https://doi.org/10.1038/s41598-018-24733-0

Salafsky N, Salzer D, Stattersfield AJ, et al (2008) A standard lexicon for biodiversity conservation: Unified classifications of threats and actions. Conserv Biol 22:897–911. https://doi.org/10.1111/j.1523-1739.2008.00937.x

Table S4. The 11 major threats recorded for each island by biogeoprahic region and provinces, as well as the total threats recorded by islands are shown. *Indicates the threats that were excluded for modeling Scenario 2.

Island Name	Biogeographic region	Biogeographic province	Wildlife explotation*	Climate change*	Energy production & Mining	Geological events*	Human intrusions & disturbance	Biological Invasions	Habitat modifications	Pollution	Urbanization	Transport corridors	Total threats
Isla Clarión, Col	Neotropical	PLP	1	1	0	1	0	1	1	0	0	0	**5**
Isla Cocinas, Jal	Neotropical	PLP	0	1	0	1	0	1	0	0	0	0	**3**
Isla El Farallon, Sin	Neotropical	PLP	0	1	1	1	0	1	0	0	0	0	**4**
Isla Grande Ixtapa, Gro	Neotropical	PLP	0	1	0	1	1	1	1	1	0	0	**6**
Isla Isabela, Nay	Neotropical	PLP	0	1	0	1	1	1	1	0	0	0	**5**
Isla La Larga, Nay	Neotropical	PLP	0	1	0	1	0	1	0	0	0	0	**3**
Isla La Redonda, Nay	Neotropical	PLP	0	1	0	1	0	1	0	0	0	0	**3**
Isla Los Patos, Sin	Neotropical	PLP	0	1	0	1	0	0	0	0	0	0	**2**
Isla Pájaros, Sin	Neotropical	PLP	1	1	0	1	0	0	0	0	0	0	**3**
Isla Roqueta, Gro	Neotropical	PLP	0	1	0	1	1	1	1	1	0	0	**6**
Isla San Ignacio, Sin	Neotropical	PLP	0	1	0	1	0	0	0	0	0	0	**2**
Isla San Pancho, Jal	Neotropical	PLP	0	1	0	1	0	1	0	0	0	0	**3**
Isla Santa María, Sin	Neotropical	PLP	0	1	0	1	0	0	0	0	0	0	**2**
Isla Socorro, Col	Neotropical	PLP	1	1	0	1	1	1	1	1	0	0	**7**
Isla Venados, Sin	Neotropical	PLP	0	1	0	1	0	0	0	0	0	0	**2**
Islote Peña Blanca, Col	Neotropical	PLP	0	1	0	1	0	0	0	0	0	0	**2**
Isla La Peña, Nay	Neotropical	PLP	0	1	0	1	0	1	0	0	0	0	**3**
Isla María Cleofas, Nay	Neotropical	PLP	0	1	0	1	0	1	0	0	0	0	**3**
Isla María Madre, Nay	Neotropical	PLP	0	1	0	1	1	1	1	1	1	0	**7**
Isla María Magdalena, Nay	Neotropical	PLP	0	1	0	1	0	1	0	0	0	0	**3**
Isla San Juanito, Nay	Neotropical	PLP	0	1	0	1	0	0	0	0	0	0	**2**
Cayo Arcas, Cam	Neotropical	YPP	0	1	0	1	0	0	0	0	0	0	**2**
Cayo Centro, QR	Neotropical	YPP	0	1	0	1	1	1	1	1	0	0	**6**
Cayo Contoy, QR	Neotropical	YPP	0	1	0	1	0	1	1	1	0	0	**5**
Cayo Culebra, QR	Neotropical	YPP	0	1	0	1	0	1	0	0	0	0	**3**
Cayo Holbox, QR	Neotropical	YPP	0	1	0	1	1	1	1	1	1	0	**7**
Cayo Lobos, QR	Neotropical	YPP	0	1	0	1	0	0	0	0	0	0	**2**
Cayo Norte, QR	Neotropical	YPP	0	1	0	1	0	1	1	1	0	0	**5**
Cayo Pérez, Yuc	Neotropical	YPP	0	1	0	1	1	1	1	0	0	0	**5**
Isla Cozumel, QR	Neotropical	YPP	1	1	0	1	1	1	1	1	1	1	**9**
Isla Mujeres, QR	Neotropical	YPP	0	1	0	1	1	1	1	1	1	1	**8**
Isla Piedra, Cam	Neotropical	YPP	0	1	0	1	0	1	0	0	0	0	**3**
Isla Techal, QR	Neotropical	YPP	0	1	0	1	0	0	0	0	0	0	**2**
Cayo Lobos, Ver	Neotropical	VP	0	1	0	1	0	1	1	1	0	0	**5**
Cayo de Enmedio, Ver	Neotropical	VP	0	1	0	1	0	1	1	1	0	0	**5**
Cayo Isla Verde, Ver	Neotropical	VP	0	1	0	1	0	1	1	0	0	0	**4**
Isla de Burros, Ver	Neotropical	VP	0	1	0	1	0	1	1	1	0	0	**5**
Isla Del Carmén, Cam	Neotropical	VP	0	1	0	1	1	1	1	1	1	1	**8**
Isla El Toro, Ver	Neotropical	VP	0	1	0	1	0	1	1	1	0	0	**5**
Isla Juana Ramírez, Ver	Neotropical	VP	0	1	0	1	0	1	1	1	1	0	**6**
Isla Pérez, Ver	Neotropical	VP	0	1	0	1	0	1	1	1	0	0	**5**
Isla Alcatraz, Son	Nearctic	SP	0	1	0	1	0	1	0	0	0	0	**3**
Isla Chapetona, Son	Nearctic	SP	0	1	0	1	0	0	0	0	0	0	**2**
Isla Coloradito, BC	Nearctic	SP	0	1	0	1	0	0	0	0	0	0	**2**
Isla El Muerto, BCS	Nearctic	SP	1	1	0	1	0	0	0	0	0	0	**3**
Isla El Pelícano, Son	Nearctic	SP	0	1	0	1	0	0	0	0	0	0	**2**
Isla Encantada, BC	Nearctic	SP	0	1	0	1	0	1	0	0	0	0	**3**
Isla Patos, Son	Nearctic	SP	0	1	0	1	0	0	0	0	0	0	**2**
Isla San Luis, BC	Nearctic	SP	0	1	0	1	0	0	0	0	0	0	**2**

Isla San Pedro Nolasco, Son	Nearctic	SP	0	1	0	1	0	0	0	0	0	0	**2**
Isla Tiburón, Son	Nearctic	SP	1	1	0	1	0	1	0	0	0	0	**4**
Isla Turners, Son	Nearctic	SP	1	1	0	1	0	0	0	0	0	0	**3**
Isla Willard, BC	Nearctic	SP	0	1	0	1	0	0	0	0	0	0	**2**
Islotes Blancos, BC	Nearctic	SP	0	1	0	1	0	0	0	0	0	0	**2**
Roca Cholludo, Son	Nearctic	SP	0	1	0	1	0	0	0	0	0	0	**2**
Isla Ángel de la Guarda, BC	Nearctic	BCP	1	1	0	1	0	1	0	0	0	0	**4**
Isla Asunción, BCS	Nearctic	BCP	0	1	0	1	1	1	0	0	0	0	**4**
Isla Ballena, BCS	Nearctic	BCP	0	1	0	1	0	0	0	0	0	0	**2**
Isla Bota, BC	Nearctic	BCP	0	1	0	1	0	0	0	0	0	0	**2**
Isla Brosa, BCS	Nearctic	BCP	0	1	0	1	0	0	0	0	0	0	**2**
Isla Cabeza de Caballo, BC	Nearctic	BCP	1	1	0	1	0	0	0	0	0	0	**3**
Isla Cardonosa Este, BC	Nearctic	BCP	0	1	0	1	0	0	0	0	0	0	**2**
Isla Cayo, BCS	Nearctic	BCP	0	1	0	1	0	0	0	0	0	0	**2**
Isla Cedros, BC	Nearctic	BCP	1	1	0	1	1	0	1	0	0	0	**5**
Isla Cerraja, BC	Nearctic	BCP	0	1	0	1	0	0	0	0	0	0	**2**
Isla Cerralvo, BCS	Nearctic	BCP	1	1	0	1	1	1	1	0	0	0	**6**
Isla Coronados, BCS	Nearctic	BCP	1	1	0	1	0	1	0	0	0	0	**4**
Isla Coyote, BCS	Nearctic	BCP	0	1	0	1	0	1	0	0	0	0	**3**
Isla Danzante, BCS	Nearctic	BCP	1	1	0	1	0	0	0	0	0	0	**3**
Isla del Carmen, BCS	Nearctic	BCP	1	1	0	1	0	0	0	0	0	0	**3**
Isla El Borrego, BC	Nearctic	BCP	0	1	0	1	0	0	0	0	0	0	**2**
Isla El Pardito, BCS	Nearctic	BCP	0	1	0	1	0	1	0	0	0	0	**3**
Isla El Requeson, BCS	Nearctic	BCP	0	1	0	1	0	1	0	0	0	0	**3**
Isla Espiritu Santo, BCS	Nearctic	BCP	1	1	0	1	0	1	0	0	0	0	**4**
Isla Estanque, BC	Nearctic	BCP	1	1	0	1	0	0	0	0	0	0	**3**
Isla Gallina, BCS	Nearctic	BCP	0	1	0	1	0	0	0	0	0	0	**2**
Isla Gallo, BCS	Nearctic	BCP	0	1	0	1	0	0	0	0	0	0	**2**
Isla Gaviota, BCS	Nearctic	BCP	0	1	0	1	0	0	0	0	0	0	**2**
Isla Granito, BC	Nearctic	BCP	0	1	0	1	0	0	0	0	0	0	**2**
Isla Islitas, BCS	Nearctic	BCP	0	1	0	1	0	0	0	0	0	0	**2**
Isla La Rasa, BC	Nearctic	BCP	0	1	0	1	0	0	0	0	0	0	**2**
Isla La Ventana, BC	Nearctic	BCP	0	1	0	1	0	0	0	0	0	0	**2**
Isla Lagartija, BC	Nearctic	BCP	0	1	0	1	0	0	0	0	0	0	**2**
Isla Las Ánimas, BCS	Nearctic	BCP	0	1	0	1	0	0	0	0	0	0	**2**
Isla Las Galeras Este, BCS	Nearctic	BCP	0	1	0	1	0	0	0	0	0	0	**2**
Isla Las Galeras Oeste, BCS	Nearctic	BCP	0	1	0	1	0	0	0	0	0	0	**2**
Isla Magdalena, BCS	Nearctic	BCP	1	1	0	1	1	0	1	0	1	0	**6**
Isla Mejia, BC	Nearctic	BCP	0	1	0	1	0	0	0	0	0	0	**2**
Isla Mitlán, BCS	Nearctic	BCP	0	1	0	1	0	0	0	0	0	0	**2**
Isla Monserrat, BCS	Nearctic	BCP	1	1	0	1	0	0	0	0	0	0	**3**
Isla Mosca, BCS	Nearctic	BCP	0	1	0	1	0	0	0	0	0	0	**2**
Isla Natividad, BCS	Nearctic	BCP	0	1	0	1	1	1	1	0	0	0	**5**
Isla Pardo, BCS	Nearctic	BCP	1	1	0	1	0	0	0	0	0	0	**3**
Isla Partida Norte, BC	Nearctic	BCP	0	1	0	1	0	0	0	0	0	0	**2**
Isla Partida, BCS	Nearctic	BCP	1	1	0	1	1	0	1	0	0	0	**5**
Isla Pata, BCS	Nearctic	BCP	0	1	0	1	0	0	0	0	0	0	**2**
Isla Pata, BCS	Nearctic	BCP	0	1	0	1	0	0	0	0	0	0	**2**
Isla Piedra, BCS	Nearctic	BCP	0	1	0	1	0	0	0	0	0	0	**2**
Isla Piojo, BC	Nearctic	BCP	1	1	0	1	0	0	0	0	0	0	**3**
Isla Roca Lobos, BC	Nearctic	BCP	0	1	0	1	0	0	0	0	0	0	**2**
Isla Salsipuedes, BC	Nearctic	BCP	1	1	0	1	0	0	0	0	0	0	**3**
Isla San Benito Este, BC	Nearctic	BCP	0	1	0	1	0	1	0	0	0	0	**3**
Isla San Benito Medio, BC	Nearctic	BCP	0	1	0	1	0	1	0	0	0	0	**3**
Isla San Benito Oeste, BC	Nearctic	BCP	0	1	0	1	1	1	1	0	0	0	**5**
Isla San Cosme, BCS	Nearctic	BCP	0	1	0	1	0	0	0	0	0	0	**2**
Isla San Damian, BCS	Nearctic	BCP	0	1	0	1	0	0	0	0	0	0	**2**

Isla San Diego, BCS	Nearctic	BCP	0	1	0	1	0	0	0	0	0	0	**2**
Isla San Esteban, Son	Nearctic	BCP	1	1	0	1	0	0	0	0	0	0	**3**
Isla San Francisco, BCS	Nearctic	BCP	1	1	0	1	0	0	0	0	0	0	**3**
Isla San Ildefonso, BCS	Nearctic	BCP	0	1	0	1	0	0	0	0	0	0	**2**
Isla San José, BCS	Nearctic	BCP	1	1	0	1	1	0	1	0	0	0	**5**
Isla San Lorenzo Norte, BC	Nearctic	BCP	0	1	0	1	0	0	0	0	0	0	**2**
Isla San Lorenzo, BC	Nearctic	BCP	1	1	0	1	0	0	0	0	0	0	**3**
Isla San Marcos, BCS	Nearctic	BCP	1	1	1	1	1	0	1	1	0	0	**7**
Isla San Pedro Martir, BC	Nearctic	BCP	1	1	0	1	0	0	0	0	0	0	**3**
Isla San Roque, BCS	Nearctic	BCP	0	1	1	1	1	1	1	1	0	0	**7**
Isla Santa Catalina, BCS	Nearctic	BCP	1	1	0	1	0	0	0	0	0	0	**3**
Isla Santa Cruz, BCS	Nearctic	BCP	1	1	0	1	0	0	0	0	0	0	**3**
Isla Santa Inez, BCS	Nearctic	BCP	0	1	0	1	0	0	0	0	0	0	**2**
Isla Santa Margarita, BCS	Nearctic	BCP	1	1	0	1	1	1	1	0	0	0	**6**
Isla Santiago, BCS	Nearctic	BCP	0	1	0	1	0	0	0	0	0	0	**2**
Isla Smith, BC	Nearctic	BCP	1	1	0	1	0	0	0	0	0	0	**3**
Isla Tijeras, BCS	Nearctic	BCP	0	1	0	1	0	0	0	0	0	0	**2**
Isla Tortuga, BC	Nearctic	BCP	0	1	0	1	0	0	0	0	0	0	**2**
Isla Coronado Medio, BC	Nearctic	CP	0	1	0	1	1	1	0	0	0	0	**4**
Isla Coronado Norte, BC	Nearctic	CP	0	1	0	1	1	1	0	0	0	0	**4**
Isla Coronado Sur, BC	Nearctic	CP	1	1	0	1	1	1	1	0	0	0	**6**
Isla San Jerónimo, BC	Nearctic	CP	0	1	0	1	1	0	1	0	0	0	**4**
Isla San Martín, BC	Nearctic	CP	0	1	0	1	1	0	1	0	0	0	**4**
Isla Todos Santos Norte, BC	Nearctic	CP	0	1	0	1	1	1	1	0	0	0	**5**
Isla Todos Santos Sur, BC	Nearctic	CP	0	1	0	1	1	1	1	0	0	0	**5**
